# Tibial hemimelia: new classification and reconstructive options

**DOI:** 10.1007/s11832-016-0785-x

**Published:** 2016-12-01

**Authors:** Dror Paley

**Affiliations:** Paley Institute, 901 45th St., West Palm Beach, FL 33407 USA

**Keywords:** Tibial hemimelia, Weber patelloplasty, Paley classification, Clubfoot, Tibial aplasia, Brown centralization of fibula

## Abstract

Tibial hemimelia is a rare congenital lower limb deficiency presenting with a wide spectrum of associated congenital anomalies, deficiencies and duplications. Reconstructive options have been limited, and the gold standard for treatment has remained amputation with prosthetic fitting. There is now a better understanding of the genetics, etiology and pathoanatomy of tibial hemimelia. Armed with this knowledge, I present here a new classification to guide treatment and prognosis and then discuss new treatment strategies and techniques for limb reconstruction based on this new classification scheme.

## Introduction

Tibial hemimelia presents with a wide range of pathology, ranging from a hypoplastic tibia to complete absence of the tibia. The fibula is always present and may be normally formed or dysplastic and, in some cases, duplicate. The quadriceps muscle may be normally formed, distally deficient or absent, and the patella may be normally formed, dysplastic or absent. Similarly, the cruciate and collateral ligaments may be present or absent. The knee may fully extend or have a flexion contracture or dislocation. The foot may be normally formed, deficient or duplicated. The ankle may range from normal motion to fixed equino-varus. Tibial hemimelia can be unilateral or bilateral, with an estimated 30% of cases being bilateral [1]. Spiegel noted that 72% of unilateral cases reported in the literature affected the right side [2]. The degree of dysplasia and type may vary significantly between sides. Unilateral cases have a leg length discrepancy.

The spectrum of pathology in tibial hemimelia is much wider than that seen with congenital femoral deficiency or fibular hemimelia. In fibular hemimelia, deficiency of rays of the foot is common but duplication is never seen. In contrast, in tibial hemimelia there can be foot ray deficiency or, more commonly, foot ray duplication. Duplication of skeletal elements is a hallmark of many cases of tibial hemimelia. This duplication can affect the toes, metatarsals, tarsals, fibula, femur and femoral condyle. At the same time partial or complete deficiency can also affect these same bones in association with tibial hemimelia.

Tibial hemimelia is associated with congenital anomalies affecting the ipsilateral limb either as suppressive or duplicated [3–7]. Associated anomalies include radial dysplasia, lobster claw deformity, hand syndactyly, polydactyly, triphalagism, missing fingers or toes, hip dysplasia, hip dislocation, coxa valga, hemivertebrae and myelomeningocele [8–11]. Other associated congenital anomalies include deafness, cleft palate, pseudo-hermaphroditism, cryptorchidism and hypospadias [1]. Schoenecker et al. reported on 57 patients with tibial hemimelia of whom 34 (60%) had other associated congenital anomalies [11]. Launois and Kuss found that 24 of 41 (59%) patients with tibial hemimelia had other congenital associated anomalies [12].

The incidence of tibial hemimelia is reported to be one per million live births [13, 14]. Parent to child transmission [15, 16] as well as families with multiple siblings affected [17] have been described. Clark [18], and Lenz [19, 20] suggested that tibial hemimelia was an autosomal dominant disorder, while autosomal recessive inheritance was described by Fried [21], Mahjlondji [22] and McKay [23]. In a breeding trial of Galloway cattle with tibial hemimelia, Ojo et al. implicated homozygosity of a single autosomal recessive gene with variable expressivity and pleiotropic effects on various body systems [24].

Tibial hemimelia is associated with several syndromes. Werner’s syndrome [25] is an autosomal dominant disorder that is currently thought to be a variant of triphalangeal thumb-polysyndactyly syndrome (TPTPS). Both diseases have been mapped to chromosome 7q [26]. A deletion on chromosome 8q, contiguous with Langer–Giedion syndrome, or type II tricho–rhino–phalangeal syndrome (TRPS II) may also be responsible for tibial hemimelia [27]. CHARGE syndrome, which is a pattern of congenital anomalies, including eye, nose, ear, heart, and genital defects, as well as tibial hemimelia [28, 29] is a mutation of the CHD7 gene (chromodomain helicase DNA-binding protein 7), located on chromosome 8q. CHD7 is known to be expressed by the developing limb bud mesenchyme [30, 31]. Tibial hemimelia is also linked to tibial hemimelia–diplopodia syndrome [32], tibial hemimelia–split hand and foot syndrome [33] and tibial hemimelia–micromelia–trigonal brachycephaly syndrome [34]. The Gollop–Wolfgang complex is a very rare malformation characterized by ectrodactyly of the hand, ipsilateral bifurcation of the femur and tibial hemimelia [35]; both autosomal dominant and recessive inheritance have been reported for this malformation [35].

At the molecular level, mutations of an enhancer of Sonic Hedge Hog (SHH) have been implicated in syndromic tibial hemimelia cases [36–38]. Diemling et al [39] recently described a deletion within the SHH repressor GLI3 in two patients with bilateral tibial hemimelia. They postulated that this leads to failure to restrict SHH signaling in the posterior aspect of the limb bud, which may cause failure of the tibia to form [39].

The pathoanatomy of limbs affected by tibial hemimelia has been examined. Evans and Smith [40] found either an absence or duplication of some muscles, with some muscles being functionless and attached to only one bone. Based on their results, these authors postulated a mesoblast disorder. Hovelacque and Noel suggested the same in their study of mouse embryos in 1909 [41]. Turker et al. [42] dissected limbs with complete tibial aplasia and consistently observed that the affected leg had a dimple where the skin was tethered over fibula. The saphenous and lesser saphenous veins and sural and superficial and deep peroneal nerves were all intact. The posterior tibial neurovascular bundle was found to be short and acting as a tether. The lateral and superficial posterior compartment muscles were intact with normal insertions. The anterior and deep posterior compartments did not have a discrete boundary and their tendons had anomalous courses and sometimes split. There were no identifiable posterior tibial or anterior tibial muscle bellies, but all specimens had a tendon inserting medially on the midfoot that tethered the foot in supination while three specimens had an anomalous tendon inserting onto the neck of the talus. All specimens had a flat tendon-like structure on the anterior border of the fibula that wrapped around and inserted on the posterior capsule of the ankle. The abductor hallucis muscle was always present, even in feet without medial rays. No discrete plantar fascia was found. All specimens had subtalar coalitions, and some had midfoot coalitions and the talus articulated with the distal medial fibula on its posterolateral side.

## Classification

**Fig. 1:**
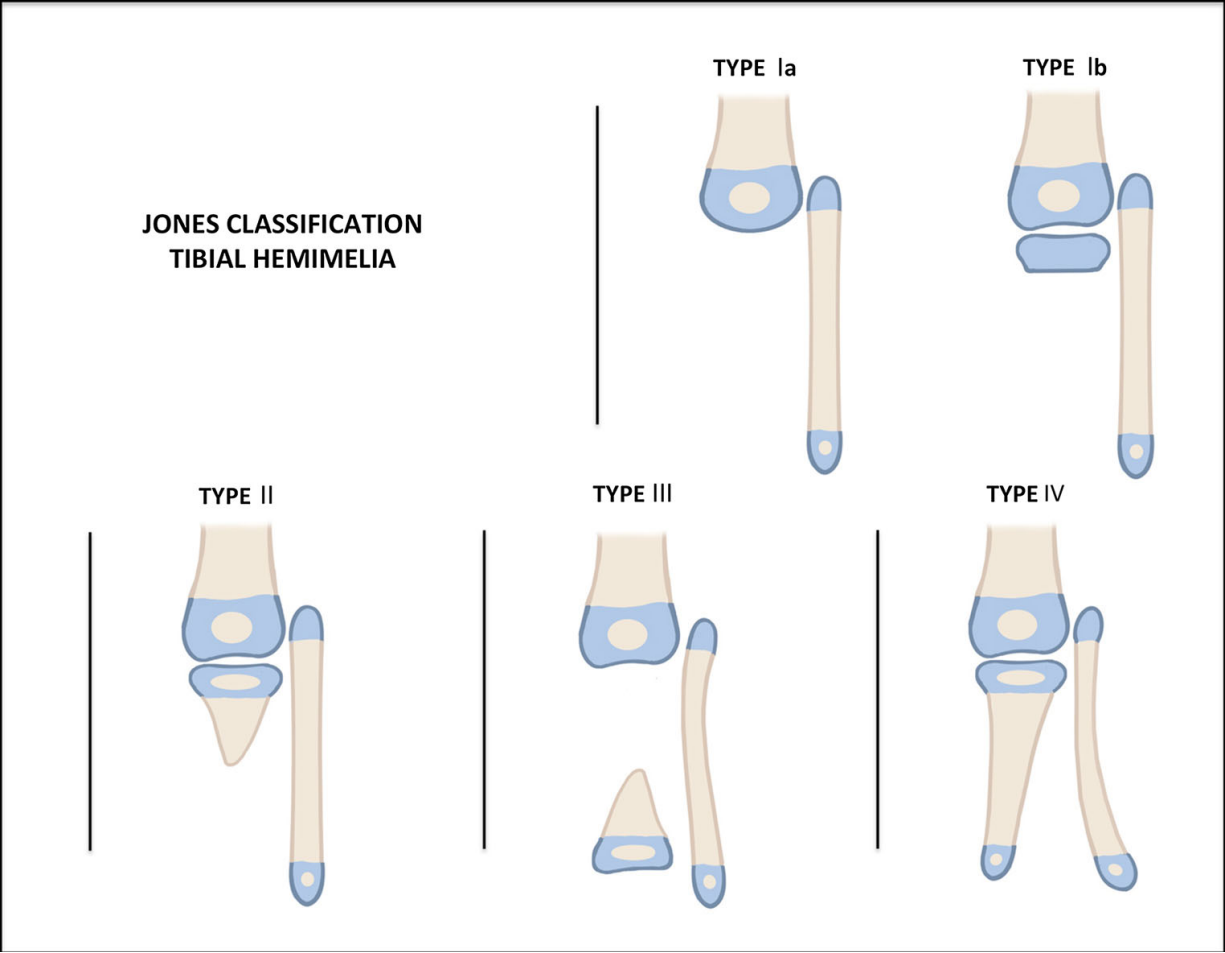
Jones classification. Reproduced with permission by the Paley Foundation

The Jones classification [43] (Fig. 1), published in 1978 and based on plain radiography findings, divides tibial hemimelia into four types, ranging from the most deficient to the least deficient. Jones type I is distinguished by the absence of a visible tibia and is subclassified into two groups: the Ia group, with a hypoplastic distal femoral epiphysis, and the Ib group, with normal ossification of the distal femoral epiphysis that suggests the presence of an unossified proximal tibial epiphysis. Jones type II condition is characterized by the presence of an ossified proximal tibia but with a distal tibia deficiency; Jones type III, by an ossified distal tibia, with a proximal tibia deficiency; Jones type IV, by a shortened tibia with distal tibio-fibular diastasis [43].

Kalamchi and Dawe modified the Jones classification eliminating Jones type III and moving Jones type Ib into type II [44]. Kalamchi type I is characterized by a total absence of the tibia, with knee flexion contracture of >45° and no active quadriceps function. The fibular head is proximally migrated with hypoplasia of the distal femur. Kalamchi type II is defined as the presence of distal tibial aplasia, with a proximal tibia present. There is active quadriceps function, with knee flexion contracture of between 25° and 45° present. There is less proximal migration of the fibula. Kalamchi type III has distal tibia aplasia with diastasis of the distal tibio-fibular syndesmosis. A normal knee joint is present and there is good quadriceps function. The talus is subluxated proximally with a prominent distal fibula.

**Fig. 2:**
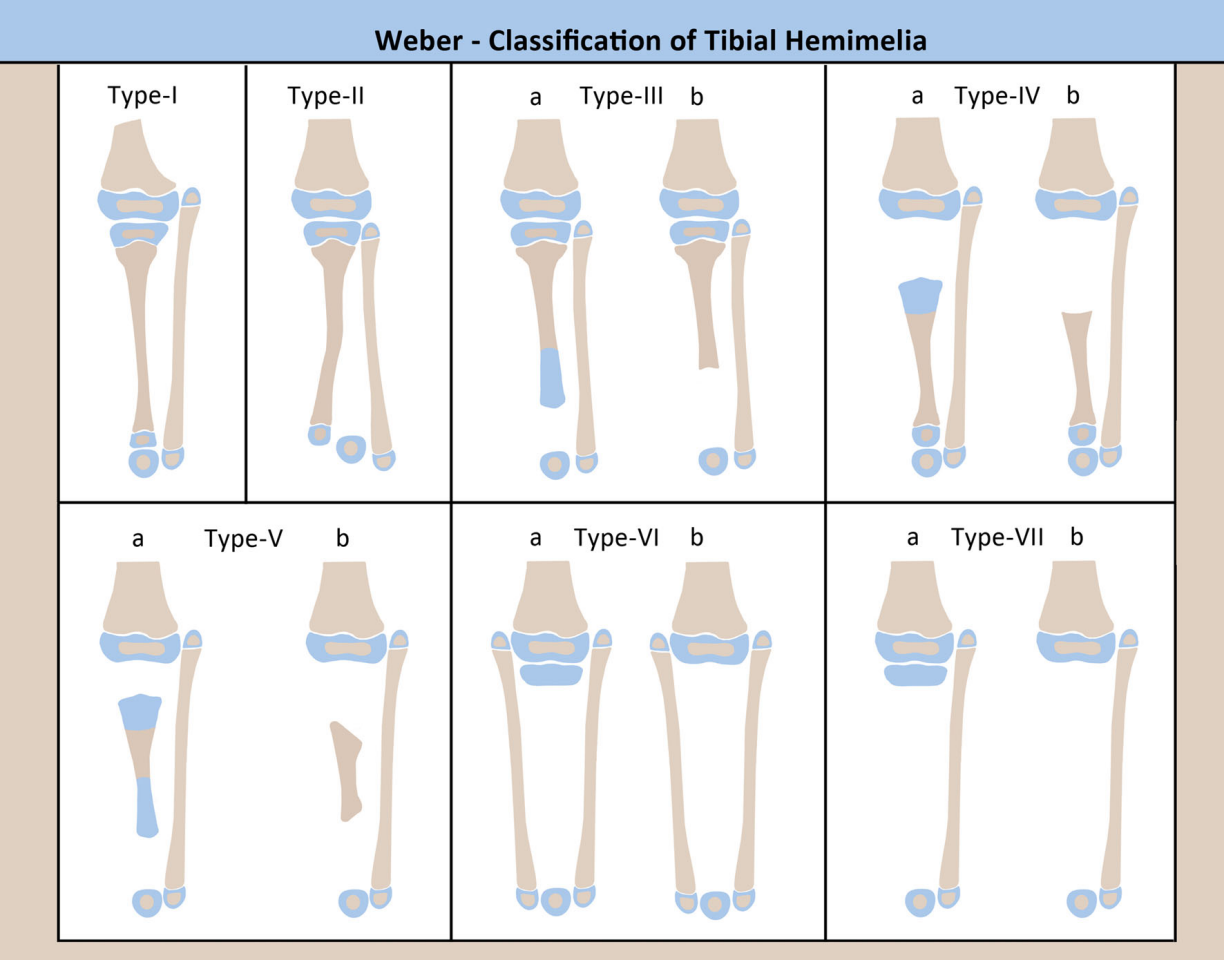
Weber classification. Reproduced with permission by the Paley Foundation

Weber [45] introduced a new classification (Fig. 2) which takes into account the cartilaginous anlage, if it is present. His classification distinguishes 7 types with 12 subtypes, including the addition of a few types of tibial hemimelia which were unclassifiable by the Jones classification [46]. Opposite to the Jones, the Weber classification is in order of increasing deficiency, with subgroups based on whether the cartilage anlage is present (a) or not (b). Weber type I is characterized by tibial hypoplasia but with intact joints proximally and distally; Weber type II, by distal diastasis of the tibia and fibula; Weber type III, by distal tibial aplasia; Weber type IV, by proximal tibial aplasia; Weber type V, by bifocal tibial aplasia proximally and distally; Weber type VI, by complete tibial agenesis with double fibulae; Weber type VII, by complete tibial agenesis with a single fibula. The Weber classification also assigns a score to determine the functional ability of the limb, taking into consideration the tibia (0–22 points), presence of an anlage (0–10 points), presence of patella (0–3 points), rest of limb (0–2 points, includes hip joint, distal femur, fibula, foot, and muscle function of hip, knee, and ankle). Higher scores indicate less impairment of the limb. Five classes are defined based on the score, indicating the degree of deficiency and difficulty of reconstruction.

**Fig. 3:**
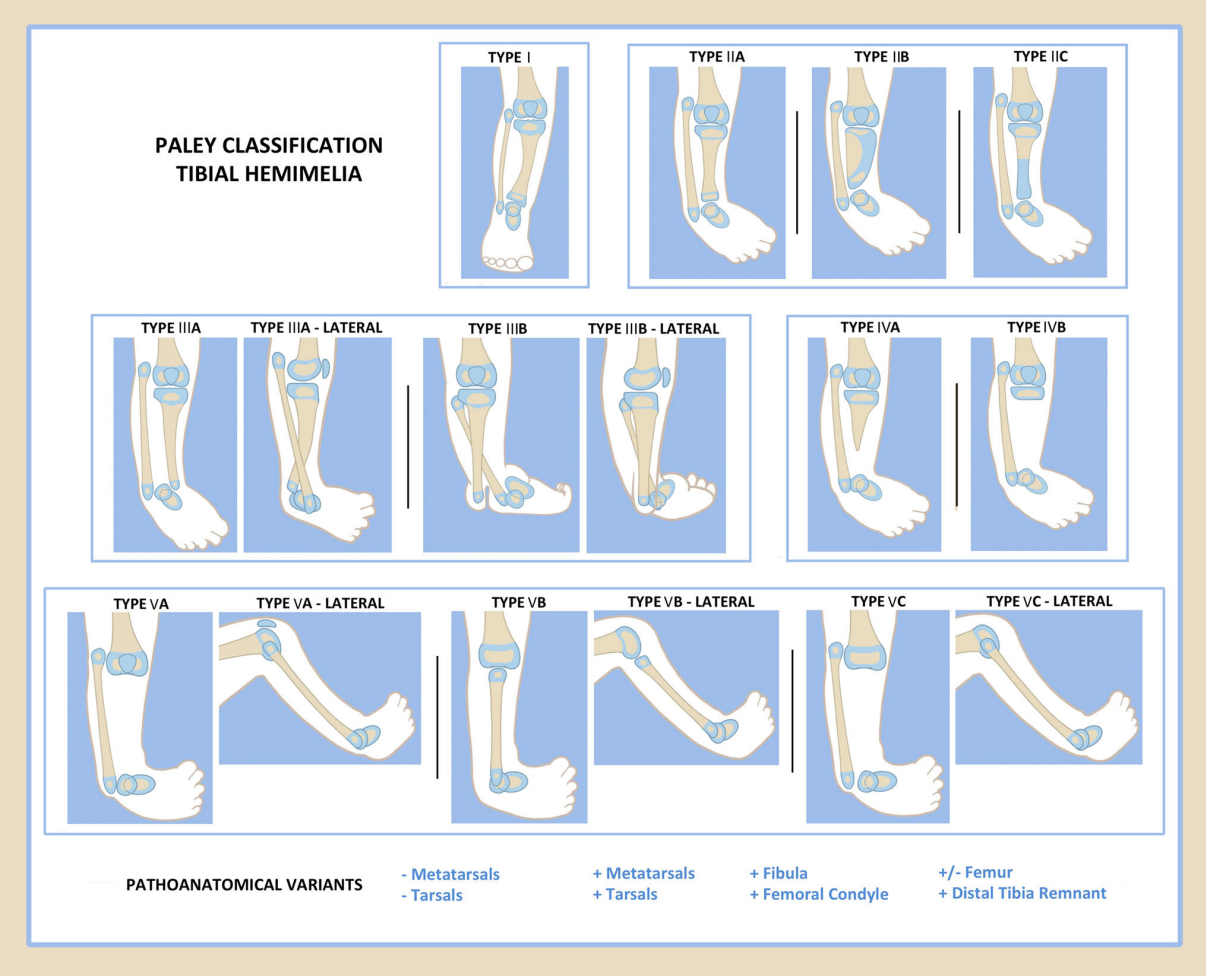
Paley classification. Reproduced with permission by the Paley Foundation

Paley proposed a new classification in 2003 [47] and modified it in 2015 [48]. This classification is being reordered one last time in this review to ensure that the level of deficiency is accurately from the least to most deficient and as such is different than that published in 2015. The Paley classification is unique in that it was developed in direct relationship to treatment and prognosis (Fig. 3). There are five types and 11 subtypes in the Paley classification, as follows.

- Paley type 1: Hypoplastic nondeficient tibia: valgus proximal tibia (genu valgum), relative overgrowth of proximal fibula, plafond present and normal
- Paley type 2: Proximal and distal tibial epiphysis present with dysplastic ankle

- Type 2A: Well-formed distal tibial physis and separate from proximal tibial physis; tibial plafond present but dysplastic; relative overgrowth of proximal fibula.
- Type 2B: Delta tibia, proximal and distal growth plates connected through bracket epiphysis, malorientation of ankle and knee joints, ankle joint dysplastic, relative overgrowth of fibula.
- Type 2C: Delayed ossification (cartilagenous anlage) of part, or all, of the tibia, dysplastic ankle joint, distal tibial physis absent, relative overgrowth of fibula.

- Paley type 3: Proximal tibia and knee joint present, medial malleolus present, distal tibial plafond absent, tibio-fibular diastasis present

- Type 3A: Tibial plafond missing, medial and lateral malleolus present, varus diaphyseal bowing tibia, distal fibula (lateral malleolus) with foot internally rotated around tibia, talus may be positioned between the tibia and fibula due to absence of tibial plafond, relative fibular overgrowth
- Type 3B: Same as 3A with skin cleft separating tibia and fibula, foot always follows the fibula

- Paley type 4: Distal tibial aplasia

- Type 4A: knee joint present, complete absence of distal tibia from level of diaphysis, pointed bone end often covered by separate skin pouch, relative overgrowth of fibula
- Type 4B: Epiphysis of proximal tibia present but absent proximal physis, knee joint present, delayed ossification of epiphysis, relative overgrowth of fibula

- Paley type 5: Complete tibial aplasia

- Type 5A: Complete absence of tibia, patella present; flexion contracture of knee, equino-varus contracture of dislocated foot and ankle
- Type 5B: Complete absence of tibia, no patella present; flexion contracture of knee, auto-centralized fibula, quadriceps present, knee capsule present
- Type 5C: Complete absence of tibia, no patella present; flexion contracture of knee, dislocated fibula, quadriceps absent, no knee capsule present.

The Paley classification also adds a modifier to describe the presence or absence of associated deficiencies and duplications. This is listed as plus (+) or minus (−) signs for toes, metatarsals, tarsals or part or all of the femur, or as + for the fibula, distal tibial remnant or femoral condyle. The wide variety of deficiencies or duplications associated with tibial hemimelia can easily be described by using the plus and minus signs with any of the types or subtypes.

Clinton and Birch [49] tried to classify 125 tibial hemimelia limbs in 95 patients treated at their institution using the Jones classification. These authors reported 73 Jones type Ia, six Jones type Ib, 18 Jones type II, zero Jones type III and 12 Jones type IV cases; there were also 16 ‘unclassifiable’ cases (12.8%). Based on these results, Clinton and Birch [49] proposed adding a Jones type 5 to represent the miscellaneous ‘unclassifiable’ cases.

Paley, Packer and Burghardt (unpublished study, presented at LLRS meeting, Charleston, SC, July 2016) recently classified 113 tibial hemimelia limbs treated earlier by the author. These authors reported 47 Jones type Ia, five Jones type 1b, 18 Jones type II, two Jones type III and 10 Jones type IV cases; 31 cases in this series were ‘unclassifiable’ (27.4%). The same series was classified by the Weber classification. There were 18 Weber type I, 11 Weber type II, three Weber type IVa, 17 Weber type IIIb, zero Weber type IVa, two Weber type IVb, five Weber type Va, zero Weber type Vb, zero Weber type VIa, zero Weber type VIb, four Weber type VIIa and 47 Weber type VIIb cases; there were also six ‘unclassifiable’ cases using the Weber classification (5.3%).

When this same group of 113 tibial hemimelia limbs was classified using the Paley classification, there were no ‘unclassifiable’ cases and no Paley types without cases. There were five Paley type 1, 11 Paley type 2A, eight Paley type 2B, four Paley type 2C, 12 Paley type 3A, six Paley type 3B, 16 Paley type 4A, four Paley type 4B, 19 Paley type 5A, five Paley type 5B, and 23 Paley type 5C cases. When the various classifications were compared, the Jones classification was by far the quickest and easiest to learn, use and memorize. In comparison, the Weber classification was very difficult to learn, use and memorize and was by far the most confusing and time consuming. The Paley classification, despite having more subtypes than the Jones classification, was still relatively easy to use, learn and memorize, as well as being relatively quick for classifying tibial hemimelia. Its + or − modifier was very helpful in conveying the picture of the associated deficiencies or duplications that were present. The ease of use of the Paley classification of tibial hemimelia, combined with its efficiency and comprehensiveness, suggest that it be proposed as the new standard.

The wide variety of pathoanatomy in tibial hemimelia does not fit perfectly into any classification scheme. One example is the case report by Shrivastava et al. of an intercalary deficiency where the central portion of the tibia is missing but the proximal and distal tibia is intact [50].

## Surgical management

Amputation is the recommended treatment for Jones type I tibial hemimelia [10, 11, 51, 52], although some authors do recommend reconstruction if the deformity is less severe [53–55], especially if there is a tibial anlage and an active quadriceps mechanism [56]. The presence of a quadriceps is inferred by the presence of a patella. Physical examination and ultrasound and magnetic resonance imaging (MRI) examinations are useful methods to determine the presence of a patella, tibial anlage and quadriceps [57].

Brown published a surgical procedure for the treatment of Jones type I by fibular centralization in 1965 [58]. This was usually combined with a Syme’s amputation. In his 15-year follow-up study (1972), in which 40 of 56 patients were available for review [59], 18 required secondary surgery due to a knee flexion deformity, 21/22 were ambulatory, and all but two were wearing braces while ambulatory. Based on the results of the follow-up study, Brown recommended attachment of the patellar ligament to the fibula, pre-operative traction, as well as femoral shortening and soft tissue releases as needed to gain extension. He also recommended surgery before age 1 year for maximal ambulatory and fibular articulation potential. Inferior results were noted with an absent quadriceps muscle.

Most authors have not reported good outcomes with the Brown procedure and have recommended knee disarticulation rather than reconstruction as the best option for total absence of the tibia [51, 52]. Many of the poor outcomes were due to progressive knee flexion contractures, knee instability and poor range of motion. For some patients in whom amputation was not an option, a limb that is weight bearing though less functional was considered a success [53].

Knee disarticulation has been described for treatment and also remains a salvage option for failed Brown procedures. Kalamachi [44] treated three children with the Brown procedure, and all went on to subsequent knee disarticulations. The failure was attributed to knee flexion contractures and the absence of active quadriceps function, leading the authors to recommend early disarticulation of the knee without any attempt of reconstruction. Alternatively, if the femur was severely hypoplastic, a femoro-fibular arthrodesis was performed to effectively lengthen the femur, creating a longer lever arm for improved prosthetic fitting. Similar results and conclusions were drawn by Schoenecker et al. [11] and Fernandez et al. [7].

Weber [46] described an innovative surgical procedure in which the patella was converted into a tibial plateau by chondrodesis to the fibula at the time of centralization. The procedure was performed in the face of the severe knee flexion contracture requiring extensive surgical release of the knee contracture. After the patellar arthroplasty was performed, the residual knee contracture together with the foot contracture were gradually distracted with an external fixator to avoid the need to shorten the femur or fibula. This was followed by a chondrodesis of the fibula to the talus. Recurrent deformity due to failure of fusion at the chondrodesis sites were common complications.

In the presence of a tibial anlage (Jones type Ib) or a proximal tibia (Jones type II), some authors report good results with tibio-fibular synostosis [44, 45]. The use of an external fixator prior to reconstruction was reported as helpful to overcome soft tissue contractures [44]. Schoenecker [11] recommended tibiofibular synostosis for Jones types Ib and II cases, combined with a Syme’s amputation. These patients were functional as below-knee amputees. Spiegel et al. [2] described some potential complications of amputation in patients with Jones type II treated with distal amputation (Chopart or Syme) due to prosthetic irritation from the overgrowth and prominence of the fibular head.

For Jones type IV deficiencies the options reported are stabilization of the ankle, arthrodesis or amputation [10, 11, 61]. Tokmakova et al. [61] felt that the treatment of choice was reconstruction of the ankle mortise as their patients were independent ambulators with stable ankles and plantigrade feet.

## The author’s approach to surgical reconstruction for tibial hemimelia

Since Brown [58] introduced centralization of the fibula, many attempts to reconstruct the knee in the most severe types (Jones I) have met with poor results, as previously discussed. Similarly, poor results of reconstruction for Jones types I, II and IV have led most surgeons to conclude that through-knee amputation for Jones type I, through- or below-knee amputation for Jones type II and Syme’s amputation for Jones type IV are the best treatment for each type of tibial hemimelia. In light of the advances in modern prosthetics, the amputation option remains the gold standard and should be considered as the most tried and proven method of treatment. However, advances in the treatment of all types of tibial hemimelia offer new surgical options with excellent functional results as an alternative to amputation. Since the Paley classification was designed as a guide to treatment and prognosis, my discussion of surgical management is presented according to Paley type and subtype.

### Paley type 1 tibial hemimelia (Fig. 4)

These patients have intact stable knees and ankles. The proximal fibula is overgrown and may articulate with the side of the femur. The ankle joint is well formed and stable. There may be valgus malalignment in the tibia. If treated when the physes are open, the valgus can be corrected using hemi-epiphysiodesis. In adulthood, the valgus can only be treated by osteotomy. If bilateral, the biggest complaint of these patients is mesomelic disproportion and short stature. If unilateral, there is a leg length discrepancy. In both cases the treatment involves simultaneous deformity correction with lengthening, either uni- or bilateral. The overgrown proximal fibula can either be pulled down to station with differential lengthening of the tibia relative to the fibula or left in place while lengthening both the tibia and fibula together the same amount. Pulling down the fibula risks creating a knee flexion contracture through tightening of the biceps tendon.

**Fig. 4:**
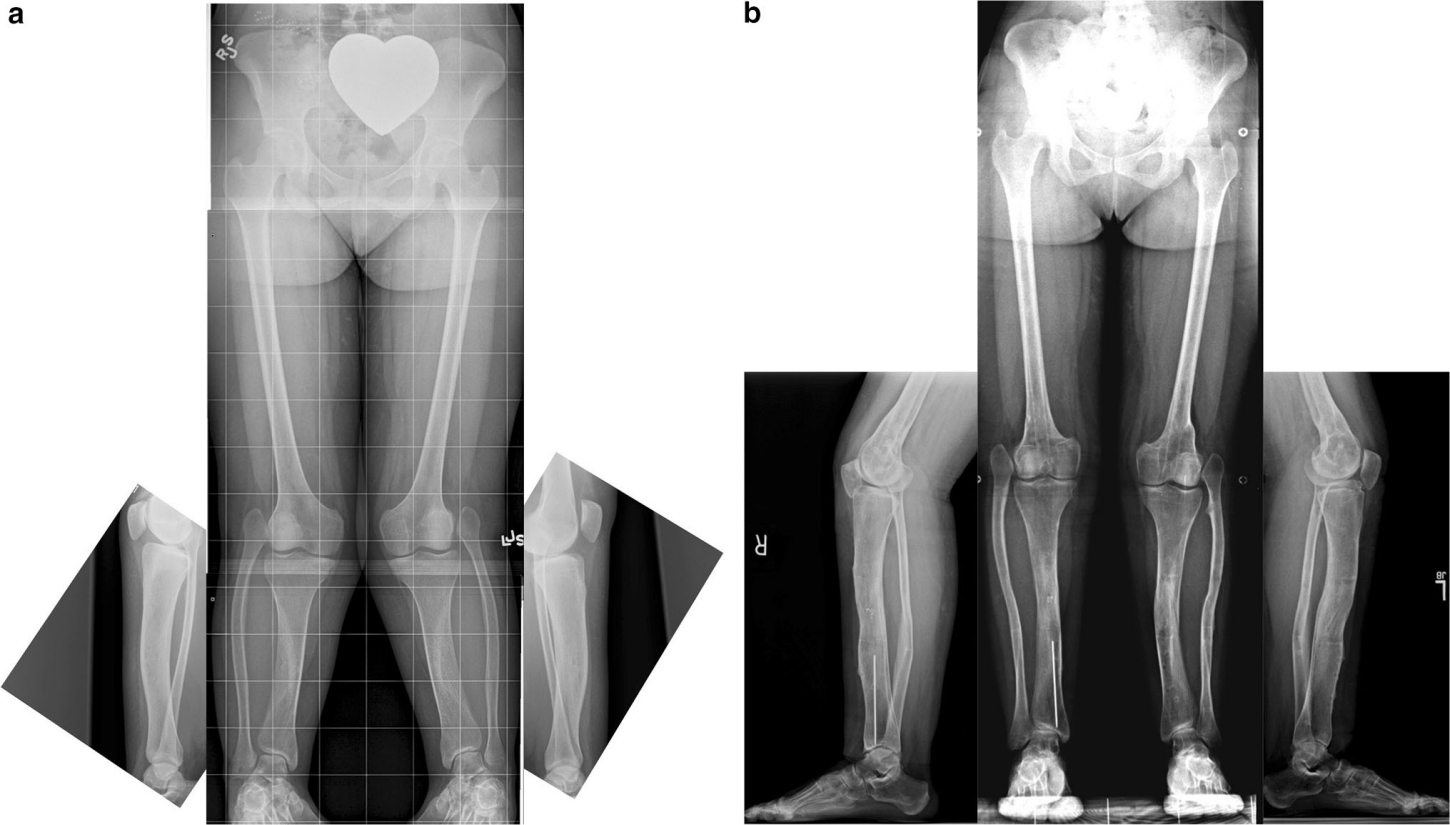
**a** Antero-posterior (AP) and lateral radiographs of 20-year-old woman with bilateral Paley type 1 tibial hemimelia. The tibia is well formed at both the knee and ankle joints. The fibulas are relatively overgrown at their proximal ends and are articulating with the side of the femurs. The knees are both in valgus due to both tibial and femoral deformities. The tibias have mild procurvatum diaphyseal bowing. Since both tibias are relatively short compared to the femurs, the patient has a mesomelic disproportion and short stature. **b** AP and lateral radiographs after treatment. Both tibias were lengthened with external fixators. The tibial valgus-procurvatum was corrected. The fibulas were not pulled down. Bilateral femur varus osteotomies were performed after completion of the tibial correction. The lengthening restored the proportion of the tibias and femurs to normal

### Paley type 2 tibial hemimelia

Patients with type 2 tibial hemimelia have a proximal and distal tibial epiphysis articulating as the knee and ankle. The knee is mobile but often unstable due to absence of cruciate ligaments and depression or deficiency of part of the tibial plateau. The ankle plafond is present but often dysplastic, and thus does not have much motion despite its presence. The presence of a plafond differentiates it from Paley type 3 tibial hemimelia which is more deficient due to the lack of a tibial plafond. Ankle diastasis is not typical, but some degree may be present depending on the severity of dysplastic changes of the tibial plafond. The foot is usually in equino-varus.

### Paley type 2A tibial hemimelia (Fig. 5)

If the foot equino-varus deformity exceeds the malorientation of the tibial plafond, which is due to bony deformity, the ankle should be distracted with an external fixator to correct the contracture followed by staged osteotomy to realign the tibia with the foot together. If the equino-varus does not exceed the tibial deformity, an osteotomy of the tibia for angular correction of the tibia with the foot, combined with lengthening, is carried out relative to the longer fibula. The circular external fixator extends from the femur to the tibia and foot.

**Fig. 5:**
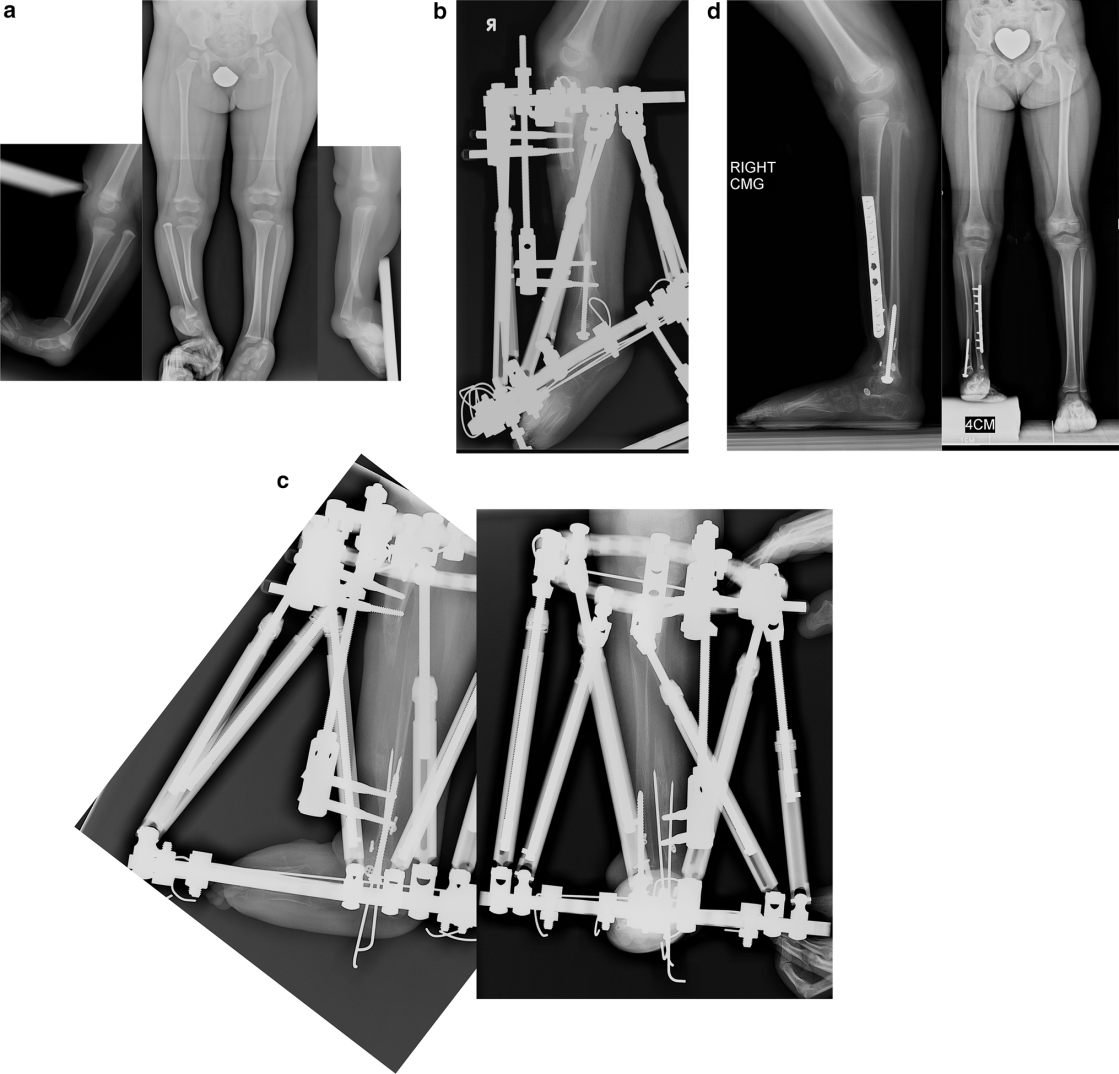
**a** Radiographs of 2-year-old girl with Paley type 2A unilateral tibial hemimelia, with equino-varus deformity of foot and varus of tibial diaphysis. There is no diastasis of the distal tibio-fibular joint. The ankle joint is present. The ossification of the distal tibia shows a regular trumpet shaped metaphyseal line indicating that this is the region of the distal tibial physis. **b** Lateral radiograph of tibia. Taylor spatial frame (TSF) is in place with proximal tibial and foot rings programmed for gradual correction of foot deformity. There is also an independent mechanism (threaded rod with cube and two half pins connected to the proximal ring) to simultaneously lengthen the tibia through a proximal osteotomy. There is a distal fibular epiphysiodesis screw in place to slow the growth of the faster growing fibula. **c** Lateral (*left*) and AP (*right*) radiographs at the end of the foot correction and 8 cm of lengthening after a second stage surgery to openly reduce the talus to the tibia combined with axial pinning of the ankle joint. Syndesmotic washer-suture device (Ziptite™ Fixation System; Biomet Sports Medicine, LLC, Warsaw, IN) inserted to stabilize the lengthened tibia to the fibula at its new syndesmosis level. The tibial lengthening bone shows poor regenerate formation. **d** Plate fixation at time of removal of fixator to prevent fracture. The tibia is well aligned. The talus is under the distal tibia. The fibula is now at station at the ankle and the ankle is stable. The foot is plantigrade

### Paley type 2B tibial hemimelia (Fig. 6)

The bracket epiphysis can be oriented in any direction and does not always correspond to the deformity seen. The fibula is much longer than the tibia. The treatment in these cases is to consider the direction of the bracket in planning the surgery. To interrupt the bracket, the cartilage of the epiphysis and physis is cut, and an osteotomy is performed through the bone at the same level. To allow for acute correction of the deformity, part of the fibula must be resected. The acute correction is accomplished by an opening wedge osteotomy on the side of the bracket, with or without a partial closing wedge on the opposite side. The correction can be done with or without lengthening at the same time. With lengthening, it is done with the external fixator extending to the femur. Without lengthening, fixation is obtained with axial retrograde wires entering through the foot and if necessary also crossing the knee.

**Fig. 6:**
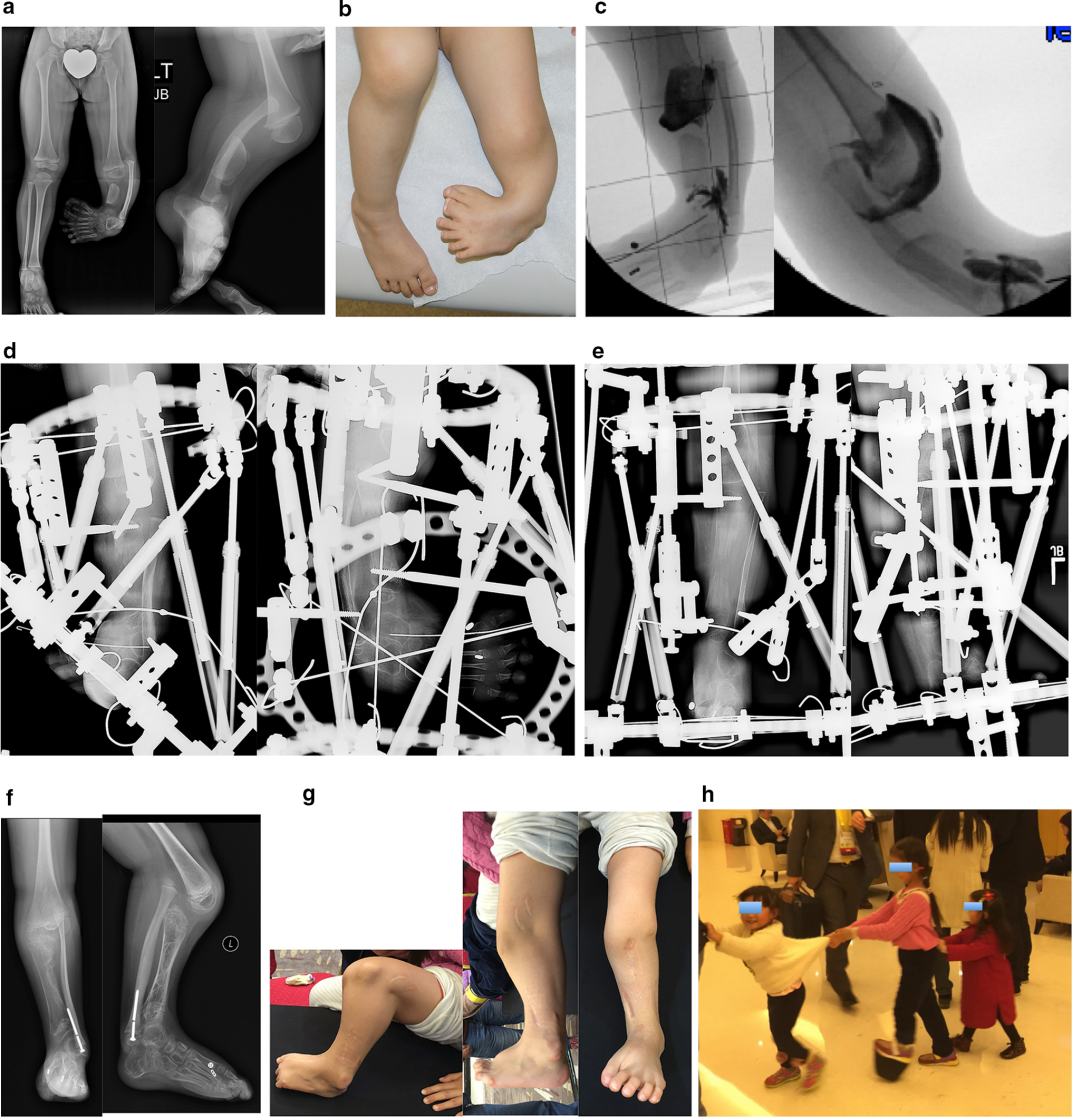
**a** Radiographs of a 3-year-old girl with unilateral tibial hemimelia, Paley type 2B tibial hemimelia + metatarsals/toes. The obvious delta tibia is seen with two secondary ossific nuclei. The foot has seven metatarsals and eight toes. The foot is in equino-varus. The ankle and knee joint are maloriented. The proximal fibula is relatively overgrown and sits proximal to the distal femoral physis. **b** Clinical photograph of same girl. **c** Arthrogram of knee and ankle showing joint malorientation and dysplasia. **d** AP (*left*) and lateral (*right*) radiographs with TSF in place and osteotomy lengthening and deformity correction of the tibia. The foot deformity correction is partially through the distal tibial reorientation. Note there is no osteotomy of the fibula and that it is only fixed to the distal tibial ring using one distal tibio–fibular wire and half pin. **e** AP (*left*) and lateral (*right*) radiographs following 8 cm of tibial lengthening and deformity correction with distal fibular transport. The frame was modified connecting the distal tibiofibular wire and fibular half pin to the proximal ring. This frees the ankle joint for distraction correction of the remaining foot deformity. Note that the proximal fibula has been pulled down almost to station. **f** Two years after removal of the external fixator the tibia is well consolidated. The fibula is already showing significant relative proximal and distal overgrowth. The distal fibular epiphysiodesis screw’s head is migrated into the epiphysis and screw shaft is broken rendering it mostly ineffective at this juncture. The foot varus deformity is slowly recurring. This will require another differential lengthening surgery in a year or two. **g** Clinical photos showing knee and ankle range of motion two years after lengthening. **h** This girl is very active despite recurrent leg length difference and mild foot deformity. Note how she plays with the other girls while she wears a shoe lift. She is the tall girl in the *middle*

### Paley type 2C tibial hemimelia (Fig. 7)

The salient feature in this type of tibial hemimelia is the delayed ossification of part, or all, of the tibia. When part of the tibia is affected, it is always the distal part. An MRI examination is useful to image the articulations between the tibia and the femur and those between the tibia and the talus. Based on these, a decision can be made as to whether the deformity of the tibia needs to be corrected in order to reorient the ankle to the knee. Since the fibula is longer than the tibia, two options can again be considered for managing the fibula: (1) resection to create a pseudoarthrosis of the fibula, or (2) lengthening of the tibia relative to the fibula. The ankle joint, while present in these cases, is not functional. The goal is to create a plantigrade foot with a stable ankle. This usually requires distraction of the foot through the ankle joint, followed by a secondary arthrotomy as described for type 2 deficiencies to best match the articulation between the talus and tibia. The syndesmosis may or may not need to be fixed with a syndesmotic suture washer device.

**Fig. 7:**
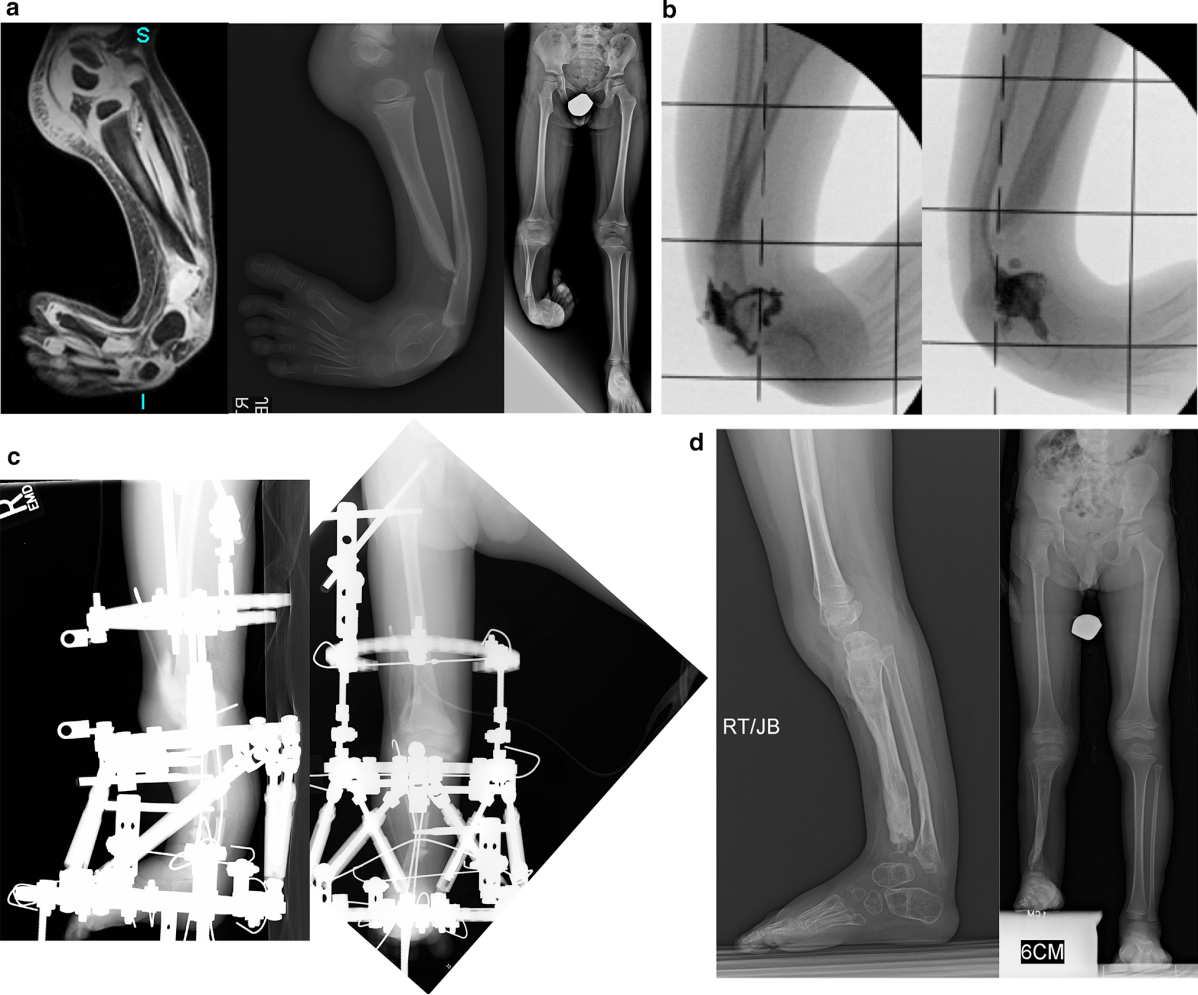
**a** Magnetic resonance imaging (MRI) scan (*left*), lateral radiograph (*middle*), and standing AP radiograph (*right*) showing Paley type 2C unilateral tibial hemimelia. The foot is in significant varus. The distal tibia has an unossified region contiguous with the ankle. There is no obvious distal tibial physis, but the plafond is present. Note that the ossification of the distal tibia is irregular and sloped, not like a normal metaphysis associated with a distal tibial physis (Fig. 5a). The proximal fibula is relatively overgrown and proximally migrated. **b** Arthrogram of ankle showing AP (*left*) and lateral (*right*) views. **c** Lateral (*left*) and AP (*right*) radiographs after arthrotomy of ankle, and open valgus-extension osteotomy and shortening of distal tibia to realign ankle joint by acute correction. Bone morphogenetic protein 2 (BMP2) was inserted in drill holes of the cartilage remnant of the distal tibia to get it to ossify. A second osteotomy was performed proximally for lengthening. The fibula is being pulled distally with an intramedullary wire hooked over the proximal epiphysis (see AP view). Because the tibial/ankle/foot deformities were corrected acutely, the external fixator is programmed for pure lengthening. The external fixator is extended to the femur with knee hinges to protect the cruciate deficient knee during lengthening while permitting knee flexion and extension motion. **d** Lateral (*left*) and AP (*right*) radiographs, after lengthening with excellent consolidation of the tibia, including ossification of the delayed distal tibia. The foot is plantigrade with forefoot supination deformity present. The proximal fibula is at station

The unossified portion of the tibia will eventually ossify after many years. To accelerate this process, bone morphogenic protein (BMP2) can be inserted into the cartilage. This is an off-label use of INFUSE (Medtronic, Memphis, TN). The basis of its use in tibial hemimelia is the author’s experience using BMP2 in delayed ossification of the femoral neck to promote ossification in congenital femoral deficiency [62]. Ossification of the tibia facilitates lengthening and deformity correction of the tibia through bone. If sufficient parts of the tibia are bony, an osteotomy can be made through the bony portion and pins placed in the bony portion. If an insufficient portion of the tibia is ossified to allow for external fixation, then open surgery is performed to acutely realign the foot with a tibial osteotomy, combined with resection of part of the fibular diaphysis. To ossify the tibial anlage (non-ossified portion of the tibia), BMP2 is inserted into drill holes in the cartilage. Stabilization of the osteotomy is achieved with retrograde axial Kirschner wires through the foot and up the tibia. In most cases, ossification of the anlage is already seen by 3 months after BMP2 implantation surgery. Lengthening is usually done 1 year later, after ossification of the unossified segment of tibia.

### Paley type 3A tibial hemimelia (Fig. 8)

The foot is in equino-varus and internally rotated relative to the knee. The talus is proximally migrated relative to the distal tibia. The talus is at the correct level relative to the distal fibula. The foot is repositioned by gradual distraction of the foot from the tibia using a circular external fixator. To prevent physiolysis of the proximal and distal fibula, a 1.5-mm wire is drilled retrograde into the fibula and up the fibular diaphysis to exit through the proximal fibular epiphysis. The wire is brought through the skin proximally and then bent backwards on itself to form a hook. A small proximal incision is made, and the wire is pulled back into the fibular head to lock into the proximal epiphysis. Distally, the wire is also bent 180°, then shortened and buried under the skin. This creates a temporary epiphysiodesis of the proximal and distal fibula to prevent physiolysis during distraction (refer to Fig. 12c for an example of this temporary epiphysiodesis wire).

**Fig. 8:**
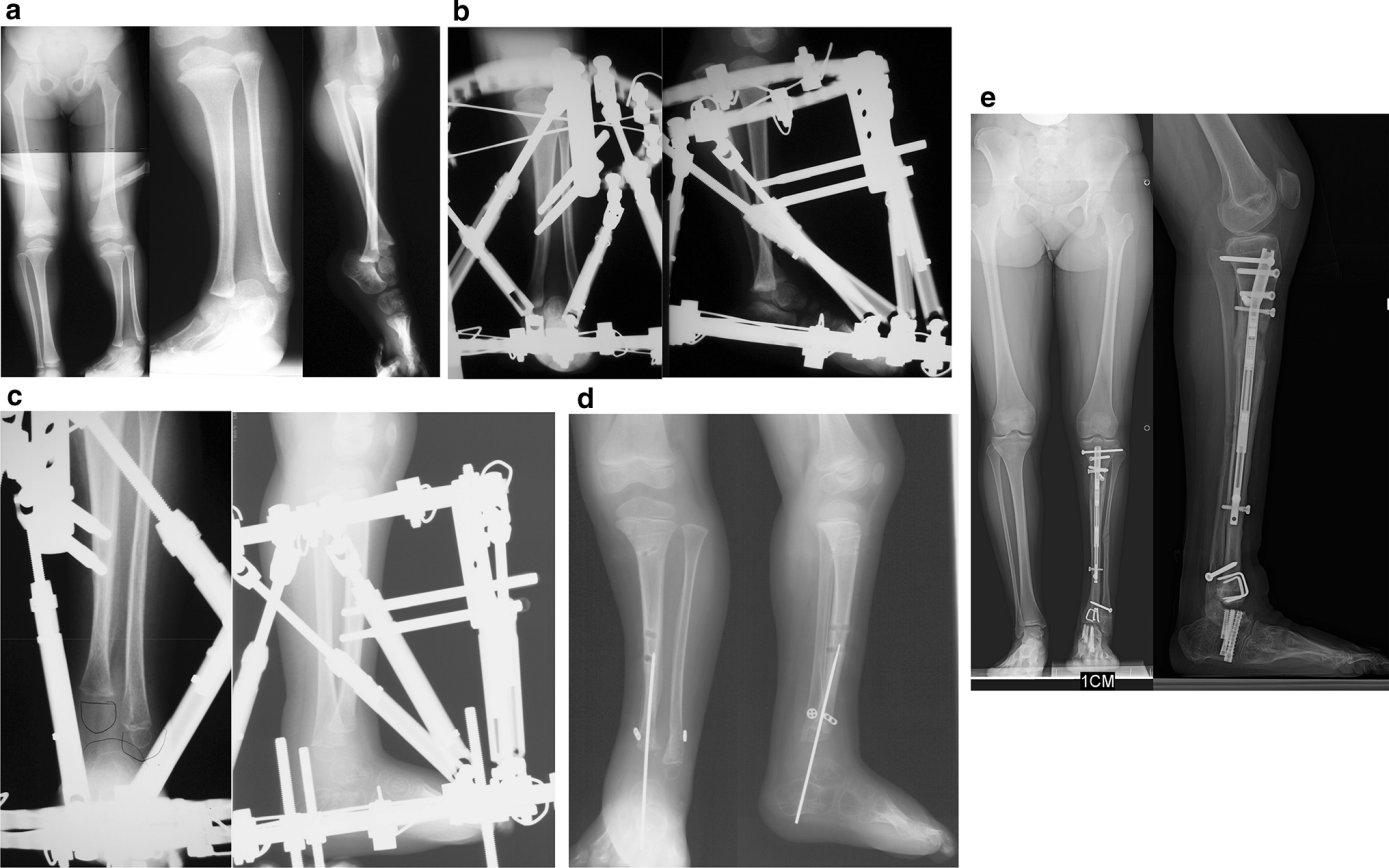
**a** Standing (*left*), mortis view (*middle*) and lateral view (*right*) radiographs of a 2-year-old girl with Paley type 3A unilateral tibial hemimelia. The diastasis of the distal tibia and fibula with the talus in between is very evident. The talus always remains in line with the distal fibula and together they internally rotate around the distal tibia. The talus is shortened relative to the distal tibia but not relative to the distal fibula. There is relative overgrowth with proximal migration of the fibula. There is a mild distal tibial varus diaphyseal bowing. **b** AP (*left*) and lateral (*right*) radiographs showing TSF applied to the tibia and foot. There is no fixation in the fibula or talus. More current constructs would fix to these as described in the text. The foot is in internal rotation and equinus. **c** AP (*left*) and lateral (*right*) radiographs after gradual correction using TSF. The talus is under the distal tibial epiphysis and the foot and fibula have rotated externally relative to the tibia. There is no longer any tibio-fibular diastasis. The equinus deformity is also corrected. This gradual correction took 12 weeks. **d** AP (*left*) and lateral (*right*) radiographs after removal of the external fixator 3 months following open ankle reconstruction. The ankle reconstruction included a biologic arthroplasty to create a concave surface on the distal tibial epiphysis that is congruent to the talar dome convexity. The ankle joint was pinned with an axial wire and the diastasis of the tibia and fibula were fixed with a suture-washer syndesmotic repair (TightRope®, Arthrex, Naples, FL). The axial wire was left in place for another 3 months to prevent recurrent deformity at the ankle. Note the proximal fibula was brought down to station. **e** AP (*left*) and lateral (*right*) radiographs at age 14 years after she underwent two successful lengthenings; one external fixator lengthening at age 6, and one implantable nail lengthening at age 14 (Precice™; NuVasive Inc., San Diego, CA). She also had a supramalleolar and subtalar osteotomy. Note the ankle joint is stable and well preserved. The ankle has about 20° of motion. The proximal fibula is at station. Most of this hardware was removed at a later date. Due to the limited ankle motion, the leg lengths were intentionally corrected to leave a 1-cm difference

One ring is applied to the proximal tibia with one wire and two half pins. The second ring is applied to the foot with three calcaneal and one talar wire (refer to Fig. 12d). The equino-varus deformity is corrected by gradual distraction, then by repositioning the talus under the distal tibial epiphysis. Since the fibula is overgrown relative to the tibia, it does not need to be fixed to the distal ring. Its association with the talus and calcaneus causes it to follow the foot distally. This moves the fibula from its relatively overgrown proximal position down to the normal station.

Once the foot is located under the distal tibial epiphysis, a planned second stage surgery can be carried out. The distal ring and wires are removed. The pin sites are covered by an occlusive dressing and the leg is prepped and draped free. A transverse incision is made on the medial side at the level of the tip of the medial malleolus. The tibio-talar joint is opened, and the distal tibia and proximal talus are exposed. The tibialis posterior tendon is located between the tibia and fibula, where the plafond should have been. It is moved out of this location to allow the fibula and tibia to come together. The tibio-fibular diastasis is treated next. This is fixed by using a syndesmotic suture system such as the TightRope® (Arthrex, Naples, FL), or the Ziptite™ Fixation System (Biomet Sports Medicine, LLC, Warsaw, IN). The syndesmotic suture with its two washers is used to reduce and compress across the diastasis.

The distal end of the tibial epiphyseal cartilage is carved with a knife to the concavity of the tibial plafond, matching the convexity of the dome of the talus, creating a biologic arthroplasty. A retrograde axial wire, perpendicular to the sole of the foot, is passed through the dome of the talus, through the epiphysis of the distal tibia, and continues proximally into the tibial intramedullary canal. If the tibia has a varus diaphyseal bow to it, a percutaneous osteotomy should be made at the apex of this bow with an acute valgus angular correction, thereby straightening the tibia. The wire is advanced up the tibia to stabilize this osteotomy.

The incision is then closed, and the foot ring is reapplied with three new wires. This helps ensure that the foot remains in a plantigrade position. The external fixator is left in place for 3 more months. The fibular wire as well as the transarticular tibial wire should be left in place even after fixator removal. The transarticular wire can be advanced into the calcaneus to allow for weight bearing. I prefer to leave both of these in place for 6 more months. This serves several purposes: prevention of fracture of the now osteoporotic tibia and fibula, stabilization of the ankle joint to prevent recurrence of equinus and retardation of the faster-growing fibula to prevent recurrent relative overgrowth. Six months later, both wires should be surgically removed. A solid Ankle-foot orthosis (AFO) is used until the wires are removed, after which the patient is placed into an articulated AFO with a plantarflexion stop. Physical therapy to regain ankle range of motion is initiated after the transarticular ankle wire is removed.

### Paley type 3B tibial hemimelia (Fig. 9)

In this type of tibial hemimelia there is a cleft between the tibia and fibula in addition to the diastasis. The first stage of treatment is the same as for type 3a tibial hemimelia. The talus is corrected out of equino-varus and brought down below the level of the distal tibial epiphysis. The second stage of surgery includes syndesmotic repair between the tibia and fibula, biologic arthroplasty of the tibio-talar joint and closure of the skin cleft.

**Fig. 9:**
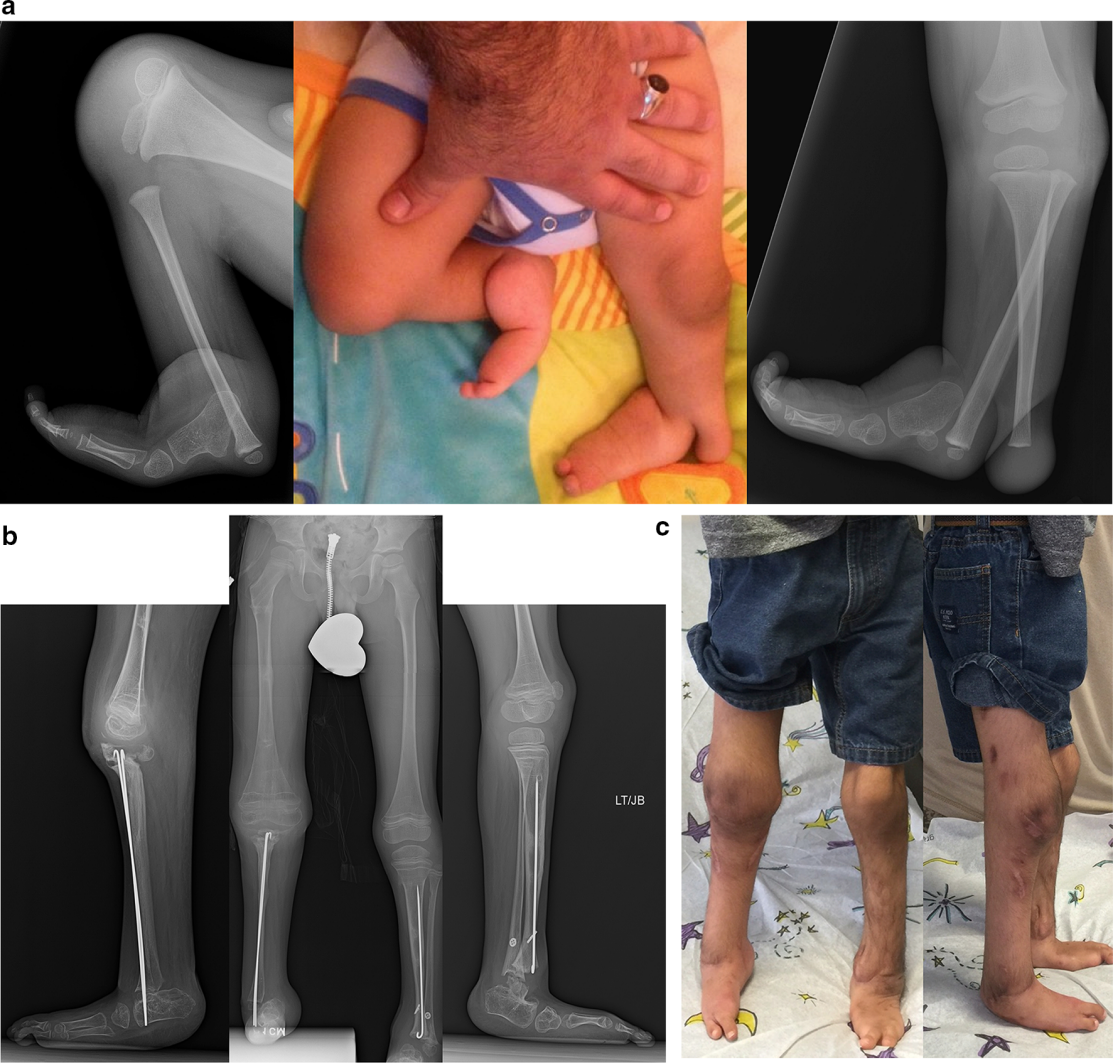
**a** Preoperative photograph (*center*), and radiographs (*right* on the *left*, *left* on the *right*) of bilateral tibial hemimelia in a 3-year-old boy; Paley type 5A (*right*) and Paley type 3B tibial hemimelia (*left*). The patella on the *right* has not yet ossified. Other than the cleft on the *left*, this is very similar to type 3A. The cleft makes this a very rare type. It should be noted that the foot is found on the fibular side of the cleft on the *left*. Both feet are in severe equino-varus and even upside down. There is a greater than 90° flexion contracture and dislocation at the right knee.** b** Standing (*center*), right lateral (*left*) and left lateral (*right*) radiographs after the staged reconstruction method was completed, 2 years prior on the *left* and 3 months prior on the *right*. The Paley–Weber patellar arthroplasty was performed on the *right*, with insertion of BMP2 to ossify the patella and fuse it to the proximal fibular epiphysis. Note the preservation of the proximal fibular physis. On the *left* the talus was brought under the tibia, a biologic arthroplasty performed together with plastic closure of the cleft. The distal and proximal tibial and fibular physes are preserved on both sides. The fusion of the talus to the distal fibular epiphysis did not occur and may require revision in the future. Both feet are plantigrade. **c** Clinical photographs showing the patient standing with both feet plantigrade and both knees straight and well aligned

### Paley type 4 tibial hemimelia 

In this type of tibial hemimelia, there is a knee joint present. The degree of deficiency of the proximal tibia varies, but the knee is present and functional. To re-establish the integrity of the tibia, the fibula is transferred to the tibia at the level of agenesis of the tibia. The foot is in very severe equino-varus. The lack of a distal tibia precludes a successful functional ankle joint. Previous attempts by the author to perform a biologic arthroplasty by various means, including by transferring the proximal fibula on its vascular pedicle, distally to the level of the ankle joint so as to create a tibial plafond, has met with recurrent deformity and failure. Fusion of the talus to the distal fibular epiphysis remains the best option.

### Paley type 4A tibial hemimelia (Fig. 10)

The proximal tibial epiphysis, physis and metaphysis are well formed in Paley type 4a tibial hemimelia. A patella is present, and active and passive knee motion is present through a normal range. The fibula is overgrown and proximally migrated, and the foot is in extreme equino-varus. The cruciate knee ligaments may be absent, and some knee instability may be present. Treatment is carried out in two stages. In the first stage the Achilles tendon is tenotomized (as is shown in Fig. 12a(iii)). Two wires are inserted into the fibula and hooked around both ends of the fibula to prevent physiolysis during distraction. These are called temporary epiphysiodesis wires. At the distal end of the fibula one of these wires is bent at 90° to be fixed to the distal tibial ring for distal transport of the fibula. A computer-dependent circular external fixator (e.g. Taylor Spatial Frame™, Smith and Nephew pic, London, UK; TL Hex™, Orthofix, Lewisville, TX; Paley Frame™, Vilex Inc., McMinnville, TN) with one ring on the femur and one ring on the tibia is applied (similar to the frame shown in Fig. 12b (iii, iv)). The knee is locked in extension by inserting one retrograde axial wire through the distal tip of the tibia up to the level of the knee joint. This tibial wire is then bent at 90° to connect to the femoral ring and hold the tibia in full extension at the knee joint. The femoral fixation includes two half-pins and one olive wire or a third half pin (as is shown in Fig. 12b(i, iii, iv)). The distal ring is fixed to three calcaneal wires and one talar wire (as is shown in Fig. 12b (ii,iii, iv)). Six struts are connected between the rings and computer planning is carried out. After surgery gradual distraction is done in two steps. In the first step the proximal fibula is realigned to correct varus-flexion deformity at the knee and transported distally to bring the fibular head down to station next to the tibia. The temporary epiphysiodesis wires inside the fibula that are hooked around the head of the fibula prevent physiolysis during this distraction. It can take 6–12 weeks to complete this first step. The second step is to gradually correct the foot equino-varus. This is done by redoing the computer planning and not by surgery. Prior to starting this second correction schedule, the fibular wire is disconnected from the distal ring and connected via a long threaded rod to the proximal ring (similar to Fig. 12c(i,ii)). This holds the fibula at station, while the foot is corrected out of its deformed position and then distracted to bring the talus below the distal fibular epiphysis (similar to Fig. 12c(iii,iv)). The foot correction usually takes another 6–12 weeks, depending on the degree of equino-varus deformity and the amount of lengthening required to transport the talus beneath the fibular epiphysis.

**Fig. 10:**
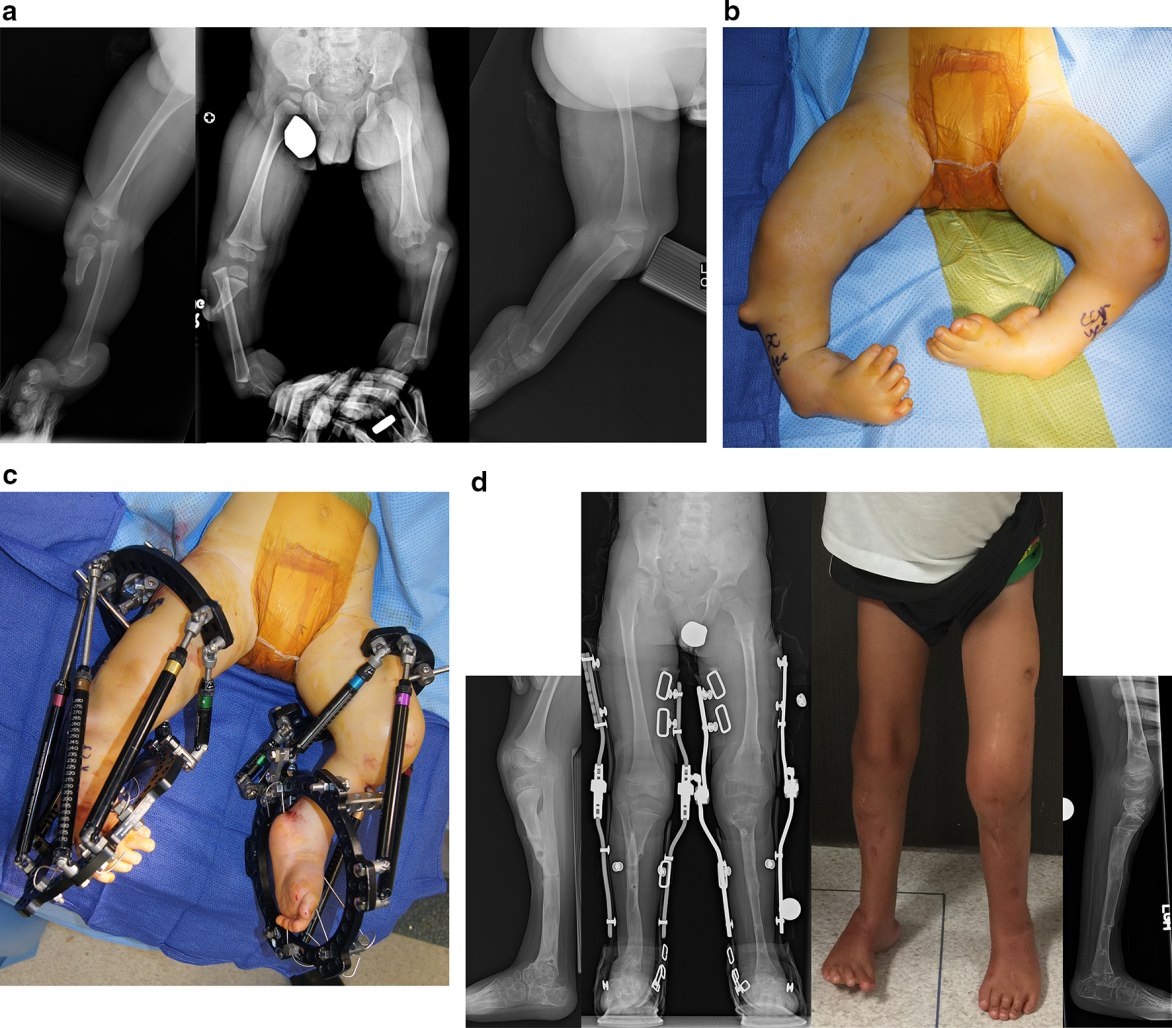
**a** Preoperative radiographs of a 13-month-old boy with bilateral tibial hemimelia; Paley type 4A tibial hemimelia on the *right* and Paley type 4B tibial hemimelia on the *left*. Both sides have severe equino-varus deformities of both feet. The proximal tibial remnant on the *right* is prominent under the skin. There is proximal migration of the fibula on both sides. There is an unossified proximal tibial cartilagenous anlage on the *left* side. **b** Clinical photograph showing the severe equino-varus feet. Note that the tip of the proximal tibial remnant has its own skin pouch. **c** Bilateral TSF devices were applied to both legs. The thigh rings are perpendicular to the femurs and the foot rings are parallel to the soles of both feet. This computer-dependent external fixator is programmed first to correct the deformities between the femur and fibula at the knee, including transporting the fibula distally. It is then reprogrammed to correct the foot deformity. No additional surgery is required to switch from knee to ankle correction. On the left leg the proximal fibular epiphysis was transported distally and brought under the tibial epiphysis. **d** Standing AP photograph (*center right*) and radiograph (*center left*) of both lower limbs at age 5 years, showing that the legs are well aligned. Lateral radiographs of right leg (*left*) and left leg (*right*) showing the feet are fused at the ankle in a plantigrade position. Both distal fibular physes are closed despite best efforts to preserve them. The right foot has some adductus supination deformity. The patient is shown after lengthening of both lower limbs was completed. The left proximal tibial epiphysis is ossified and fused to the proximal fibular epiphysis. The unossified proximal tibial epiphysis was ossified by insertion of BMP2 into drill holes in the cartilage of the tibial anlage. The ossification of the tibial epiphysis and fusion to the fibula occurred within 3 months. Note, there is preservation of the proximal physis of the fibula. On the *right* the fibula was transferred to the tibia. It has auto-bridged across to the proximal fibula. Initially, knee–ankle–foot–orthotic (KAFO) braces were used. The knee was stable enough to discontinue these after these radiographs

Once the foot is plantigrade and the talus is under the fibula, a second surgery to fuse the ankle and transfer the fibula to the tibia is performed. A few days before this surgery, the only tibial wire is removed to allow its pin site to heal prior to surgery. The foot ring and wires are removed (similar to Fig. 12d (i)). The pin sites are covered with an occlusive dressing (Tegaderm; 3M, Maplewood, MN) to minimize contamination during surgery. After the leg is prepped and draped free, a transverse lateral incision is made over the distal tip of the fibula (similar to Fig. 12f (i)). The distal epiphysis of the fibula and the dome of the talus are exposed. The capsular connections between them are cut to mobilize both bones relative to each other. A small incision is made proximally over the fibular wire. The fibular epiphysiodesis wires are cut proximally and pulled out distally. Two new wires are immediately inserted in the same track to protect the fibula from fracture. The fibula is osteoporotic at this stage, and without an intramedullary wire it could easily fracture due to manipulation that can occur during surgery. These two wires are brought out proximally through the small incision made over the head of the fibula. The distal fibular epiphysis is cut across its ossific nucleus. The talar ossific nucleus is exposed by cutting across the dome of the talus parallel to the sole of the foot (similar to Fig. 12f (i,ii)). The two ossific nuclei are then aligned, and the proximal wires are advanced through the talus and out the sole of the foot to hold the foot plantigrade to the fibula (similar to Fig. 12f(iii,iv)).

A Z-shaped incision is made around accessory skin pouch at the end of the tibia. The proximal longitudinal limb of the Z is medial to the tip of the tibia, the transverse part is in the crease below the tip and the longitudinal distal limb is lateral to the tibia. Fasciotomy of the anterior compartment is carried out. The tip of the tibial bone is uncovered. The anterior compartment muscles are elevated off of the lateral aspect of the tibia, and an extra-periosteal path is dissected to the fibula along the interosseous membrane. The fibula is exposed subperiosteally. The tibia is osteotomized near its tip to create a fresh surface for union to the transferred fibula. The wires in the fibula are pulled back to the level of the planned osteotomy. The fibula osteotomy is made at the level of the tibial cut. The fibula is then shifted over to the tibia under the muscles. The fibula is fixed to the tibia by first advancing the intramedullary wires. The fibula is then plated to the tibia using a mini locking plate and screws. All of the incisions are now closed in layers. External fixation wires are reinserted in the foot. These wires are fixed and tensioned to a ring. Six struts are connected between the femoral ring to the foot ring. The external fixator maintains the alignment of the foot and knee to achieve fusion of the tibia and fibula proximally and of the fibula and talus distally. A transverse fibular wire is added to compress the ankle fusion site. Ankle fusion takes approximately 3 months. After that, the external fixator is removed, leaving one wire buried in the foot and fibula to protect the fibula from fracture. The knee motion is restored with physical therapy. In the future, lengthening of the one bone leg can be carried out without crossing the knee joint. If symptomatic instability of the knee joint arises from the congenital absence of cruciate ligaments, the knee joint ligaments can be reconstructed.

### Paley type 4b tibial hemimelia (Fig. 10)

In this type of tibial hemimelia, there is only a proximal tibial epiphysis present and no proximal tibial physis. The proximal tibial epiphysis is often unossified at an early age. The foot is in severe equinovarus, and the fibula is relatively overgrown and proximally migrated at the knee. The treatment preferred is also a two-stage surgery similar to that described for Paley type 4a tibial hemimelia. Since the tibial epiphysis is so small it needs to be fixed to the proximal femoral ring with an axial wire and/or a transverse tibial epiphysis wire to prevent the tibial epiphysis from being transported distally during the distal transport of the proximal fibula. There are two options for the fibular transport. The first option is to bring it down to station and then osteotomize as described for type 4a. The second option is to distract the fibular head below the level of the proximal tibial epiphysis. In this case the proximal tibial epiphysis is fused to the proximal fibular epiphysis (Fig. 10left leg). This has the advantage of preserving and transferring the proximal fibular physis to become the proximal tibial physis, thus reducing leg length discrepancy from the absence of a proximal tibial physis.

Since the proximal tibial epiphysis is too small for plating, intramedullary hooked wires as shown for Paley type 5a tibial hemimelia can be used instead (similar to Fig. 12h, i). If the proximal tibial epiphysis is unossified, then BMP2 is inserted into drill holes in the epiphyseal cartilage. The fixator (similar to Fig. 12i (i,ii)) remains in place for approximately 3–4 months until the proximal fibula fuses to the tibial epiphysis proximally and the distal fibular epiphysis fuses to the talus distally. After fixator removal, a cast is used for 1 month, followed by a knee–ankle–foot orthotic (KAFO). Knee range of motion is restored with physical therapy, including active and passive range-of-motion exercises.

### Paley type 5 tibial hemimelia

Complete absence of the tibia presents the biggest challenge for reconstruction because there is no knee joint. While ankle fusion gives good function with little disability, knee fusion leads to significant disability for sitting and climbing stairs. It is preferable to avoid a knee fusion. Even if active knee motion cannot be achieved, a mobile knee joint supported by a brace is preferable to a knee fusion. This is not dissimilar to a paralytic knee from polio. Therefore, the following methods have been developed to reconstruct the knee.

### Paley type 5A tibial hemimelia (Fig. 11)

If a patella is present it can be moved to become a tibial plateau. This original concept was first published by Weber [60, 63]. The patella is moved on a vascular pedicle (called *visor flaps*) from its normal position, anterior to the femur, to the distal end of the femur. The fibula is centralized to the patella and its epiphysis fused to the patella. This procedure is referred to as the Weber patellar arthroplasty or Weber procedure.

**Fig. 11:**
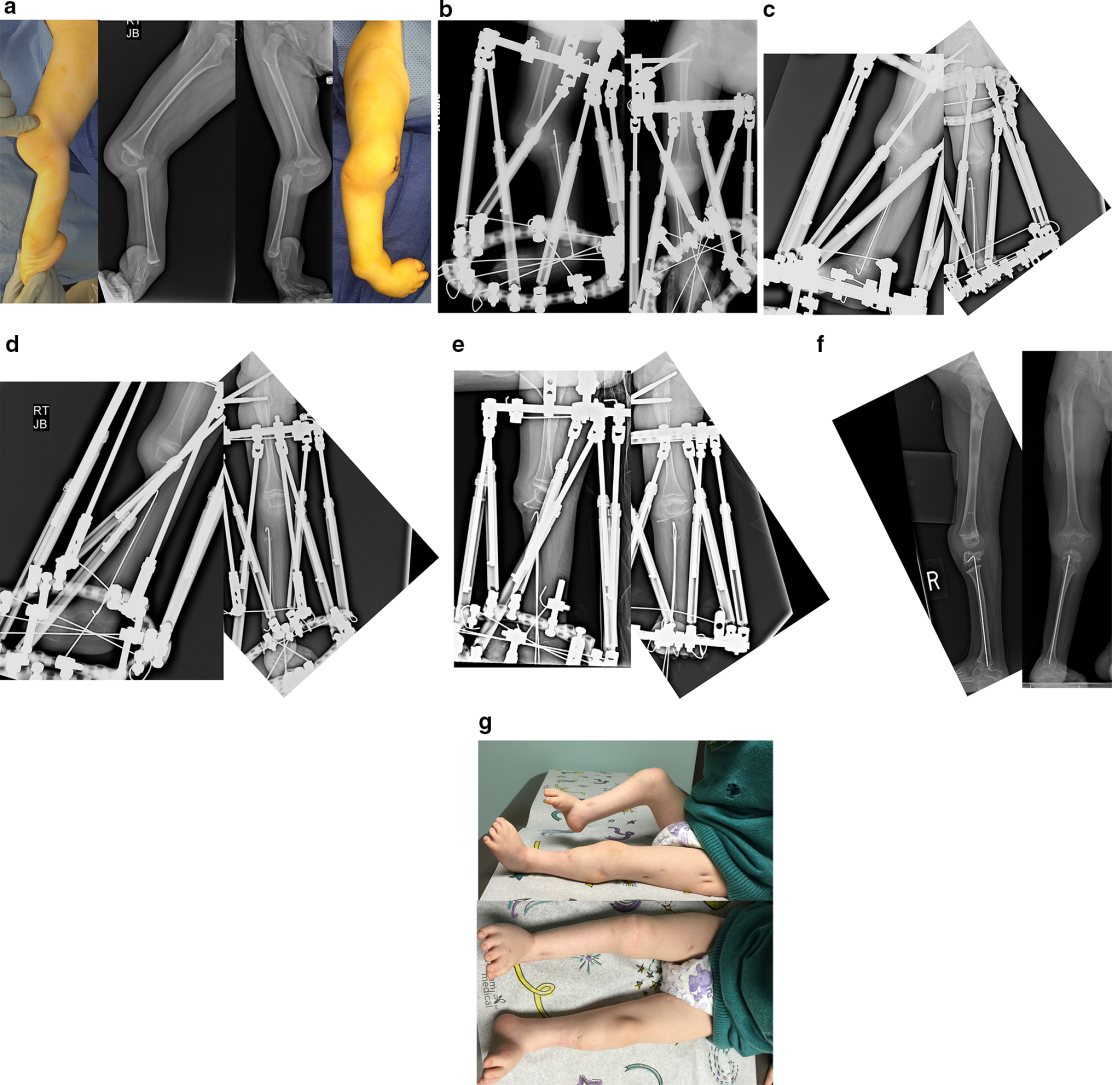
**a** Lateral (*left* and *center left*) and AP (*right* and *center right*) photographs and radiographs of the right leg of a 2-year-old boy with bilateral tibial hemimelia. Only the *right* side is shown. The *right* side has Paley type 5A tibial hemimelia and the *left* side Paley type 4A. Note the flexion contracture of the right knee and the equino-varus-adductus foot deformity. **b** Lateral (*left*) and AP (*right*) radiographs showing the TSF in place with femoral and foot rings. The fibula is secured to the foot ring with a transverse wire. Note the temporary epiphysiodesis wire in the fibula hooked at both ends. Also note the wire across the neck of the talus. **c** Lateral (*left*) and AP (*right*) radiographs at the end of gradual distraction with TSF. The fibular head is centered under the end of the femur. The foot position has not changed and the transverse fibular wires remains connected to the foot ring. **d** Lateral (*left*) and AP (*right*) radiographs showing that the transverse fibular wire was fixed to the proximal ring using a threaded rod and post. This wire was released from the distal ring to allow gradual correction of the equino-varus foot contracture and to bring the talus under the distal fibula. A second computer planning is carried out to generate a new adjustment schedule for the patient. Note that there is no ossification of the proximal fibular epiphysis. Also note the growth lines related to preoperative infusion of zolidronic acid to prevent disuse osteoporosis during distraction. **e** Intraoperative lateral (*left*) and AP (*right*) radiographs after the surgery to perform a patellar arthroplasty and fibula–talar fusion is completed. Note the hemovac drain can be seen at the knee. The Paley–Weber patellar arthroplasty was performed fixing the proximal fibular epiphysis to the patella with a hooked intramedullary wire secured to the bottom of the frame below the foot. The talus was fused to the distal tibial epiphysis. BMP2 was inserted into a drill hole in the patella and proximal fibula to lead to ossification and fusion of both the patella and fibular epiphysis. A transverse distal fibular wire is arced to the foot ring to apply compression across the fusion site. **f** Lateral (*left*) and AP (*right*) radiographs showing the patella and proximal fibular epiphysis are ossified and fused together with preservation of the proximal fibula physis. The patella now serves as a tibial plateau. The talus has also fused to the distal fibula but the distal fibular physis has closed. **g** Clinical photographs, frontal view (*bottom*) and medial view (*top*) showing that the right knee can bend to 90° (*top*). There is active motion present. The other leg was also successfully treated for type 4A tibial hemimelia

Weber recommended performing the patellar arthroplasty as the index procedure combined with gradual correction of the remaining knee flexion contracture and foot equino-varus, using a circular external fixator (Ilizarov; Smith&Nephew, London, UK). Weber performed the patellar arthroplasty procedure through a longitudinal anterior incision. Fusion of the patella to the fibula was achieved using chondroplasty by suturing perichondral flaps of the patella and fibula together. A biologic arthroplasty of the ankle was carried out to stabilize the foot.

**Fig. 12:**
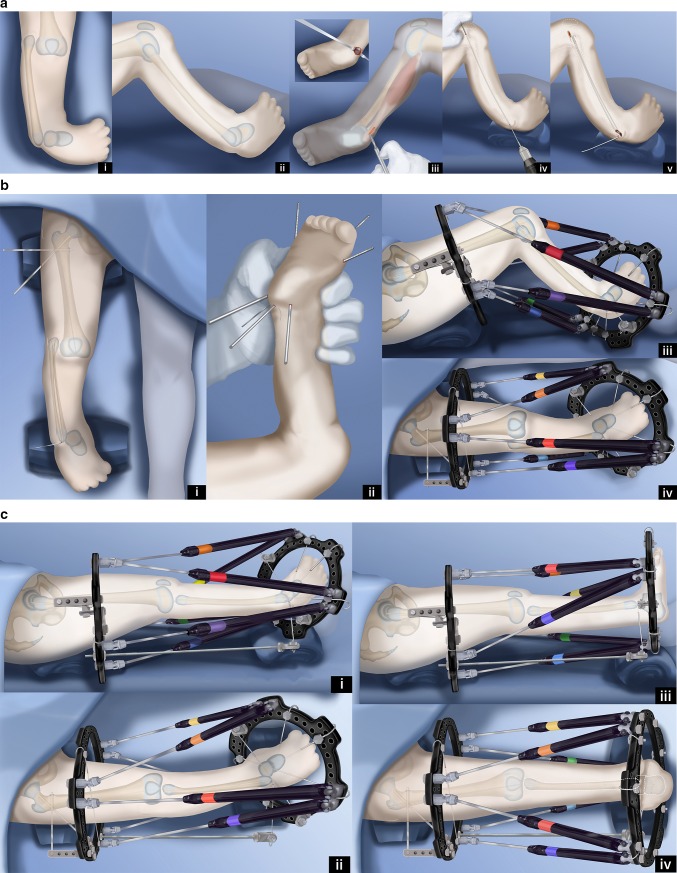

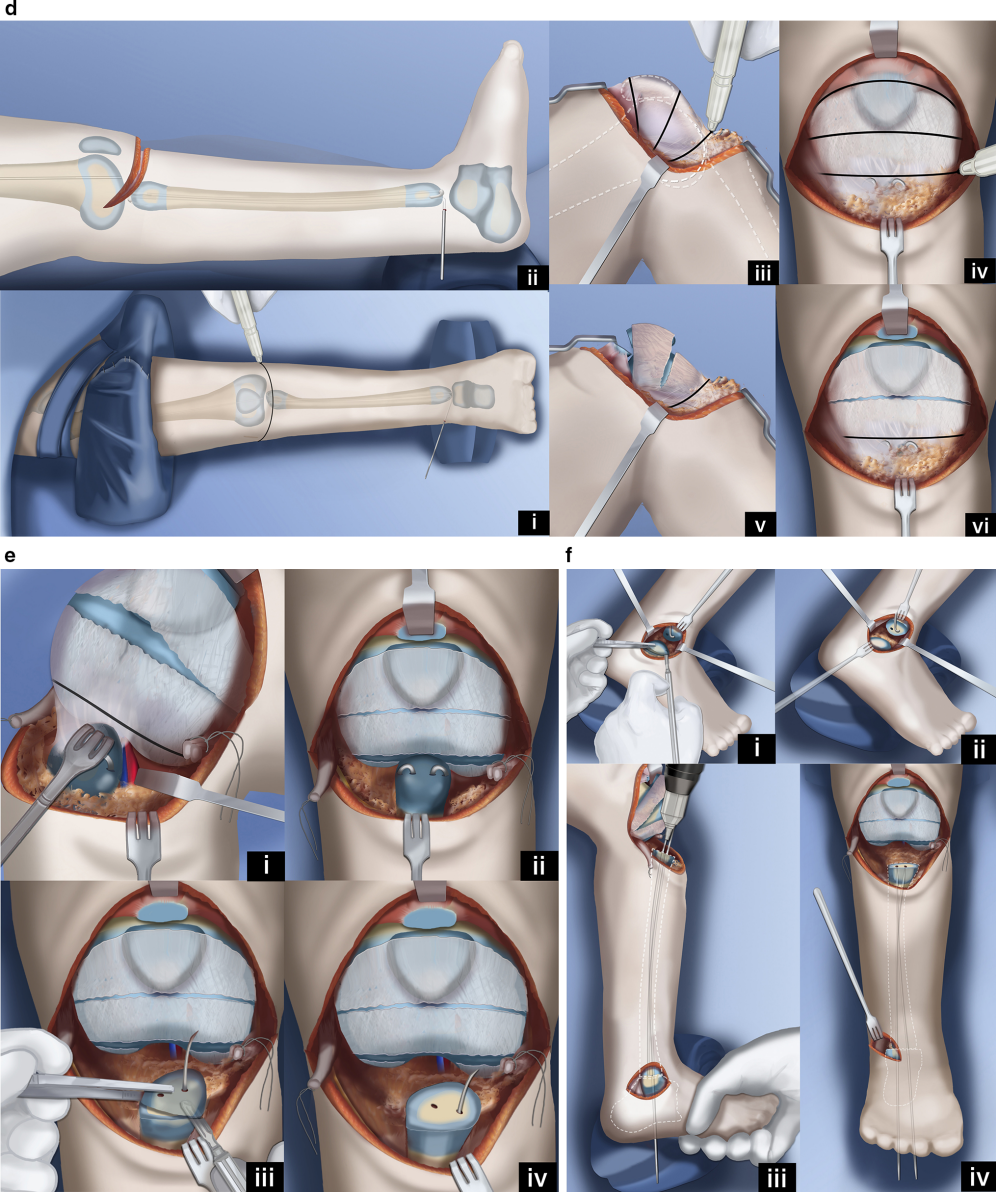

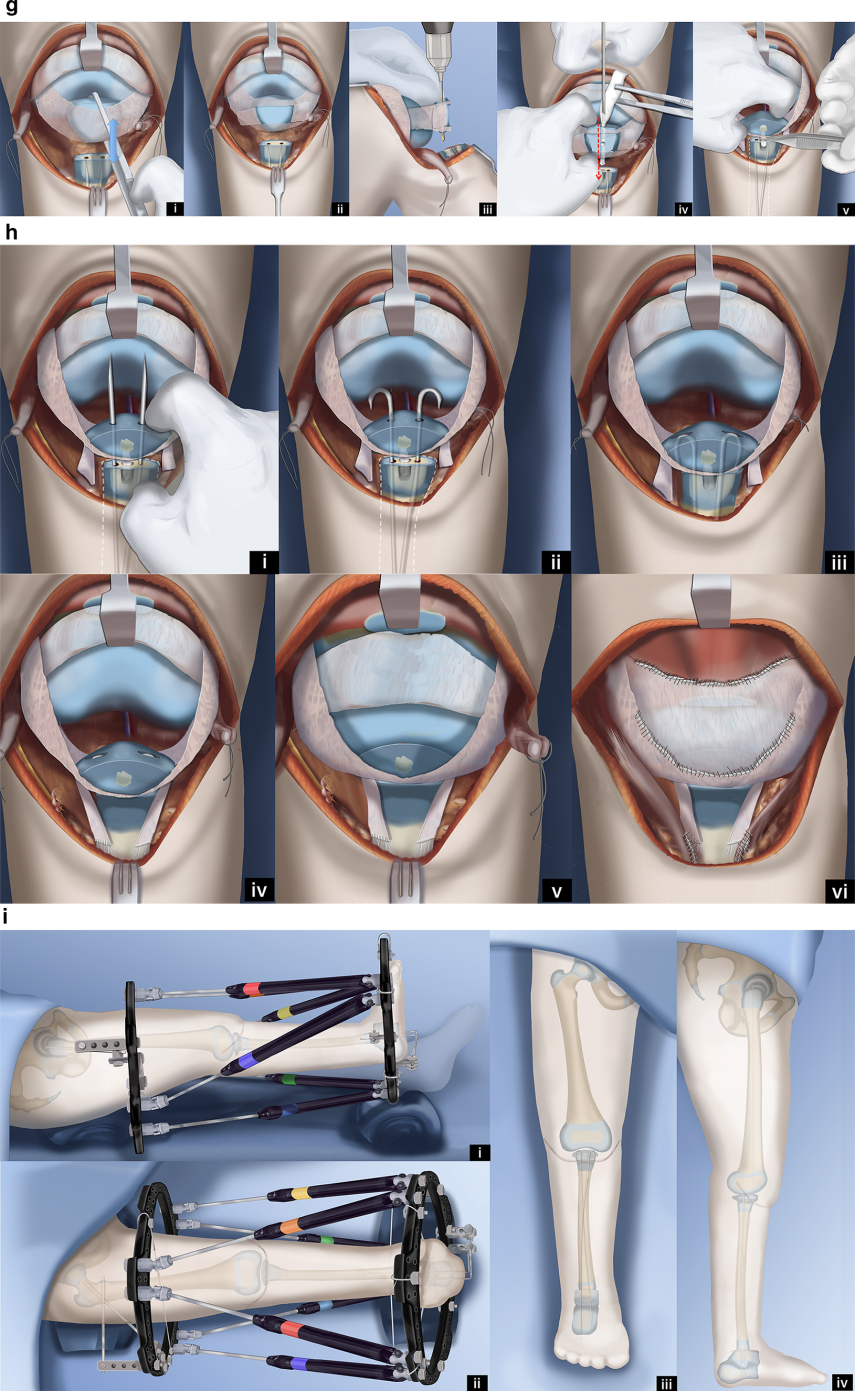
Paley–Weber patellar arthroplasty. **a** Frontal (*i*) and lateral (*ii*) view illustrations of Paley type 5A tibial hemimelia: complete tibial agenesis, patella present, fibula dislocated and proximally migrated, knee flexion contracture and fixed equino-varus foot deformity. First surgery consists of Achilles tenotomy (*iii*). Two 1.5-mm intramedullary wires are inserted retrograde into the fibula and both are curled and hooked into the proximal fibular epiphysis. One is curled around the distal fibular epiphysis and the other is bent 90° to stick out of the skin (*iv*,* v*). Reproduced with permission by the Paley Foundation. **b** A prophylactic wire is inserted in the femur and hooked over the greater trochanter. Two proximal femoral half pins are inserted—one at the level of the lesser trochanter and one up the femoral neck (*i*). Three wires are inserted in the calcaneus in the plane of the sole of the foot—one mid posterior to exit near the interspace of the first and second toes; one posterolateral to exit anteromedial; one posteromedial to exit anterolateral (*ii*). The proximal femoral ring is fixed to the two half pins and an olive wire is added from antero-medial to postero-lateral in the mid femur. A foot ring is fixed to the three wires in the foot and a transverse talar wire added. The 90° bent fibular wire is fixed to the foot ring. Finally, six struts are added between the rings. Computer planning is done to gradually correct the knee deformity (*iii*,* iv*). Reproduced with permission by the Paley Foundation. **c** After several weeks of gradual correction of the knee joint contracture, the fibula is centered on the femur. At this point the fibular wire is connected to a long threaded rod with post and then liberated from the foot ring (*i*,* ii*). A new computer planning is carried out to gradually correct the foot deformity. The talus is repositioned under the end of the fibula. The knee and ankle are now ready for the next stage surgery (*iii*,* iv*). Reproduced with permission by the Paley Foundation. **d** Second surgery starts with removal of the foot ring and wires. The leg and proximal ring are prepped and draped, and the proximal ring is covered with a sterile towel. An Esmarch is used as a tourniquet. A transverse concave proximal incision is made at the level of the knee joint (*i*,* ii*) and the knee joint capsule exposed. Two “visor” flaps should be outlined with three lines that should converge near the lateral and medial aspects of the knee. The upper line passes across the top end of the patella and the middle line at the lower end of the patella. The most distal line passes as posteriorly as possible (*iii*,* iv*). The superior pole of the patella is cut through as part of the upper incision, and a second incision is then made below the lower end of the patella (*v*,* vi*). Reproduced with permission by the Paley Foundation. **e** Before the distal part of the lower visor flap is incised, the biceps tendon is detached laterally and the semitendinosis tendon medially. The lateral border of the medial head of the gastrocnemius is located, and the popliteal vessels are identified (*i*). Staying clear of the vessels cut the posterior limb of the inferior visor flap (*ii*). The hook in the fibular wires is unfolded and one wire is removed. The epiphysis of the proximal fibula is cut through to expose its ossific nucleus (*iii*,* iv*). Reproduced with permission by the Paley Foundation. **f** A transverse incision is made at the lateral aspect of the tip of the lateral malleolus and the distal fibula and talus exposed. One fibular wire is removed and the second uncurled. The talus is cut across parallel to the sole of the foot and the distal fibula is cut across perpendicular to the fibula, exposing the bone of both ossific nuclei in preparation for fibula-talar fusion (*i*,* ii*). Remove the remaining fibular wire and replace the previous wires with two new wires. These wires are advanced through the fibula and drilled across into the talus and out the plantar aspect of the foot to fuse the fibula to the talus (*iii*,* iv*). Reproduced with permission by the Paley Foundation. **g** Slide the proximal visor flap which contains the patella posteriorly, underneath the distal visor flap, which moves anteriorly (*i*). The anterior surface of the cartilagenous patella is exposed by reflecting back two perichondral flaps (H flap;* ii*). A small hole is drilled in the patella from proximal to distal and a second hole is drilled to intersect the first in a T-junction from anterior towards posterior (*iii*). The articular surface must not be penetrated. A small shallow hole is also drilled in the epiphysis of the fibula but not deep enough to reach the physis. A BMP2 sponge (INFUSE® Bone Graft; Medtronic, Dublin, Ireland) is inserted into the holes to induce ossification of the patella and patella–fibular fusion (*iv*). The anterior and posterior holes in the patella are plugged with bone wax to prevent leakage of the BMP2 (*v*). Reproduced with permission by the Paley Foundation. **h** The retrograde fibular wires are advanced through the patella (*i*), then bent over themselves and the hook pulled back into the patella (*ii*). The apex of the bend must be submerged in the cartilage of the patella (*iii*). The perichondral flaps are sutured to the medial and lateral sides of the fibula (*iv*). The patella now sits conformed and congruent with the distal femur to act as a tibial plateau (*v*). The visor flaps are sutured together. The quadriceps muscle and patellar remnant are sutured to the proximal edge of the superior visor flap, and the the biceps and semitendinosis tendons are sutured to the lateral and medial aspects of the fibula, respectively (*vi*). Reproduced with permission by the Paley Foundation. **i** After the incision is closed layer by layer, three wires are inserted in the foot, a foot ring applied and six struts connected. One transverse distal fibular wire is arched and tensioned to compress the ankle fusion. The axial wires are fixed below to the under surface of the distal ring. The foot is plantigrade and the knee is well centered and aligned. The frame stays on the leg another 3–4 months (*i*,* ii*). After the external fixator is removed the hook wires are left in place and cut short to stay buried in the calcaneus (*iii*,* iv*). Reproduced with permission by the Paley Foundation

Paley modified the Weber procedure (Fig. 12) [48]. This Paley–Weber modification involves eight major changes to the original Weber procedure: (1) first applying a computer dependent external fixator to the femur, fibula and foot, to sequentially and gradually reposition the fibula under the femur and the talus under the fibula; (2) protecting the fibula during this distraction with temporary epiphysiodesis wires to prevent fibular physiolysis; (3) using a transverse anterior incision across the knee joint to minimize wound complications; (4) dividing the upper pole of the patella when creating the upper visor flap to allow a patella to regenerate; (5) using hooked wires for stabilization of the patella-fibular fusion site; (6) ossifying the unossified patella in patients under age 4 by inserting BMP2 into drill holes in the patella; (7) fusing the ossific nucleus of the proximal fibula to the patella with the aid of the BMP2; (8) fusion of the distal fibular epiphysis to the talus through a transverse lateral incision at the ankle.

### Paley–Weber patellar arthroplasty (Fig. 12)

A transverse concave proximal incision is made over the distal femur and proximal fibula. The fibula, patella and distal femur are exposed. Three lines outlining two visor flaps (like the visor on a motor cycle helmet) are marked. At the medial and lateral ends, the pedicle for each visor is kept as wide as possible. The proximal visor flap contains the patella. The distal visor flap is all capsular. In the Paley modification, the quadriceps muscle remains attached to the superior pole of the patella and no Z lengthening is done to the quadriceps tendon. This allows a new patella to form anterior to the femur. The proximal two visor incisions are made. The most inferior one is made after first detaching the biceps tendon laterally and the semitendinosis tendon medially. The medial head of the gastrocnemius muscle is identified. Dissection is carried out along the lateral border of this muscle to identify and protect the popliteal vessels. Once the vessels are protected, the inferior visor capsular incision is made. The perichondrium on the anterior surface of the patella is incised like the capital letter H, creating two flaps of perichondrium. The superior visor flap is brought under the inferior one to move it distally. If the patella is unossified, it is drilled in a T-shaped fashion to insert BMP2. Bone wax is used to seal the anterior and posterior aspect of the drill hole in the patella after the BMP2 is inserted in order to prevent leakage. The proximal fibular wires are exposed. The distal fibular wires are then exposed through a transverse incision at the tip of the distal fibular epiphysis. The dome of the talus and the distal fibula are exposed with the wires in place. The wires from the fibula are unbent and removed. New wires are immediately inserted. It is important to keep intramedullary wires in the fibula to prevent inadvertent fracture of the now osteoporotic fibula. The wires are retracted distally and the proximal epiphysis is cut across with a knife and then retracted proximally so that the distal epiphysis can be cut across. The fibular epiphyseal cuts expose the ossific nuclei of the proximal and distal fibula. The talus is cut across parallel to the sole of the foot exposing the talar ossific nucleus. The two wires are withdrawn proximally to align the distal epiphysis of the fibula with the ossific nucleus of the talus. The wires are then drilled antegrade through the talus and out the plantar aspect of the foot with the foot held 90° to the fibula. These wires are then withdrawn into the fibula. The upper end of the fibula can be slightly drilled to allow for insertion of the BMP2 with the patella apposed to the fibula anterior drill hole in the patella opposed to the hole in the fibular epiphysis. The two wires exiting the foot are now advanced in a retrograde fashion through the patella, and the wires protruding on the patellar articular surface are bent 180° into a hook. The hook is advanced into the substance of the patella to pull the patella down to the fibula. These two wires need to be pulled below the articular surface to prevent their protrusion into the knee joint. The medial and lateral perichondral flaps are sutured to the side of the fibula. The visor flaps can now be sewn across to each other. At the junction of the quadriceps muscle the remnant of the patella and the muscle are sutured to the inferior visor flap, that was flipped upwards. The remnant of the patella is also sutured to this capsular flap. The inferior aspect of this capsular flap is sutured to the superior edge of the patella in its new position. The inferior aspect of the patella, which is now posterior, is sutured to the posterior capsule. The biceps and semitendinosis tendons are sutured to the lateral and medial aspects of the fibula respectively. The skin is closed in layers over a drain. The wires in the foot can be reinserted. The distal ring is fixed and tensioned to these three wires. The proximal and distal rings are connected with struts. A transverse wire is drilled into the distal fibula and then arced and tensioned to the foot ring, which compresses the ankle fusion site. The two hook wires exiting the plantar aspect of the foot are secured to the foot ring under slight tension.

The external fixator remains on for 3–4 more months. After the external fixator is removed, the patient goes into a cast for a month; when the cast is removed they are fitted for a hip–knee–ankle–foot–orthotic (HKAFO). The brace is reduced from an HKAFO to a KAFO, after a couple years. The KAFO is needed for many years until the knee is sufficiently stable to allow walking without the brace. This is usually after age 10 years, when adequate hypertrophy of the joint surface and fibula has occurred.

### Paley type 5B tibial hemimelia (Fig. 13)

If there is no patella, but the fibula is auto-centralized, then there is usually a quadriceps muscle in continuity to the fibula with a capsule present. The distal femur is usually less dysplastic in these cases. The knee still presents with a fixed flexion contracture and the foot presents as with Paley type 5A tibial hemimelia, i.e. dislocated and in extreme varus and equinus. The treatment is to distract the knee contracture until the fibula and femur are collinear with each other. The foot should also be distracted relative to the fibula to centralize it under the distal end of the fibula. This is again accomplished with a computer-dependent circular external fixator. Once the distraction correction at both the knee and ankle are completed, a second stage surgery is performed to reconstruct collateral ligaments at the knee and to advance the quadriceps muscles onto the fibula. Local tissue or allograft tendon may be used. An ankle fusion as described previously is performed. The after-treatment is with an HKAFO as described above.

**Fig. 13:**
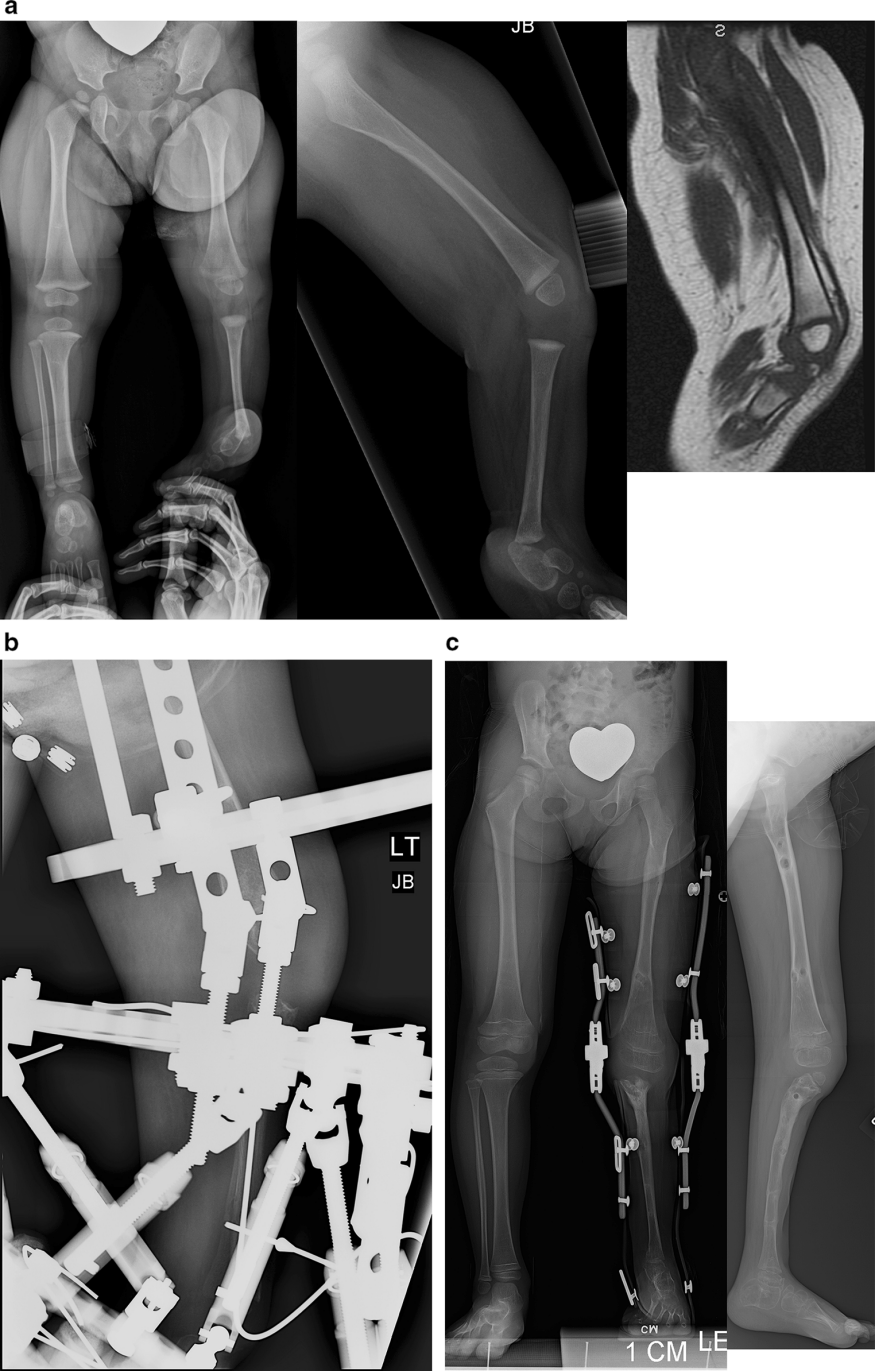
**a** Antero-posterior (*left*) and lateral (*middle*) radiographs of a 12-month-old girl with Paley type 5B unilateral tibial hemimelia. The fibula is hypertrophied and centered on the femur. The foot is in equinovarus. A sagittal section of the magnetic resonance imaging scan (*right*) shows an absent patella, with a quadriceps muscle extending to the fibula. **b** Lateral radiograph showing a TSF device with six struts applied between the upper fibula and foot to correct the equino-varus foot deformity and to bring the talus under the end of the fibula. An Ilizarov apparatus on the femur connects to the TSF with hinges at the knee joint. A distraction mechanism is in place to correct the knee contracture while permitting removal of this mechanism to allow physical therapy to move and exercise the knee through the hinges. **c** Standing AP (*left*) and lateral (*right*) radiographs showing the fibula is centralized, hypertrophied and lengthened. A KAFO brace is used to protect the stability of the knee and promote hypertrophy. The talus is fused to the fibula with the foot in a plantigrade position. The distal and proximal fibular physes are both patent

## Type 5C tibial hemimelia (Fig. 14)

If there is no patella and the fibula is dislocated, the fibula can be centralized by distraction, as was done for Paley type 5A tibial hemimelia. An Achilles tendon tenotomy is carried out. Then, two temporary epiphysiodesis wires are inserted up the fibula and hooked over the proximal epiphysis. One wire is also hooked over the distal epiphysis, while the distal end of one of the two axial fibular wires is bent 90° and brought out laterally to be fixed to the foot ring initially and later to be connected to the femoral ring. This is to protect both proximal and distal fibular physes from physiolysis during distraction. Then, one proximal and one distal circular fixator ring is applied with two half pins in the proximal femur (one up the femoral neck and one transverse at the level of the lesser trochanter) and one wire on the proximal ring in the mid-femur. A distal ring on the foot is applied, with three anterior posterior wires in the foot (one from posterior midcalcaneus to the anterior mid-forefoot, one from posteromedial calcaneus to the anterolateral forefoot, and one from the posterolateral calcaneus to the anteromedial forefoot) and one transverse wire across the ossific nucleus of the talus. Six struts are connected between the femoral and the foot ring. The computer-dependent external fixator planning is done relative to the proximal ring for the knee joint contractures. Once the fibula is reduced at the knee and the knee flexion contracture eliminated, the fibular wire is connected to the proximal ring and the computer planning repeated for the distal ring and foot contractures. When both ends of the fibula are aligned to the femur and tibia, the second stage surgery can be performed. This is referred to as the Paley knee reconstruction [48].

**Fig. 14:**
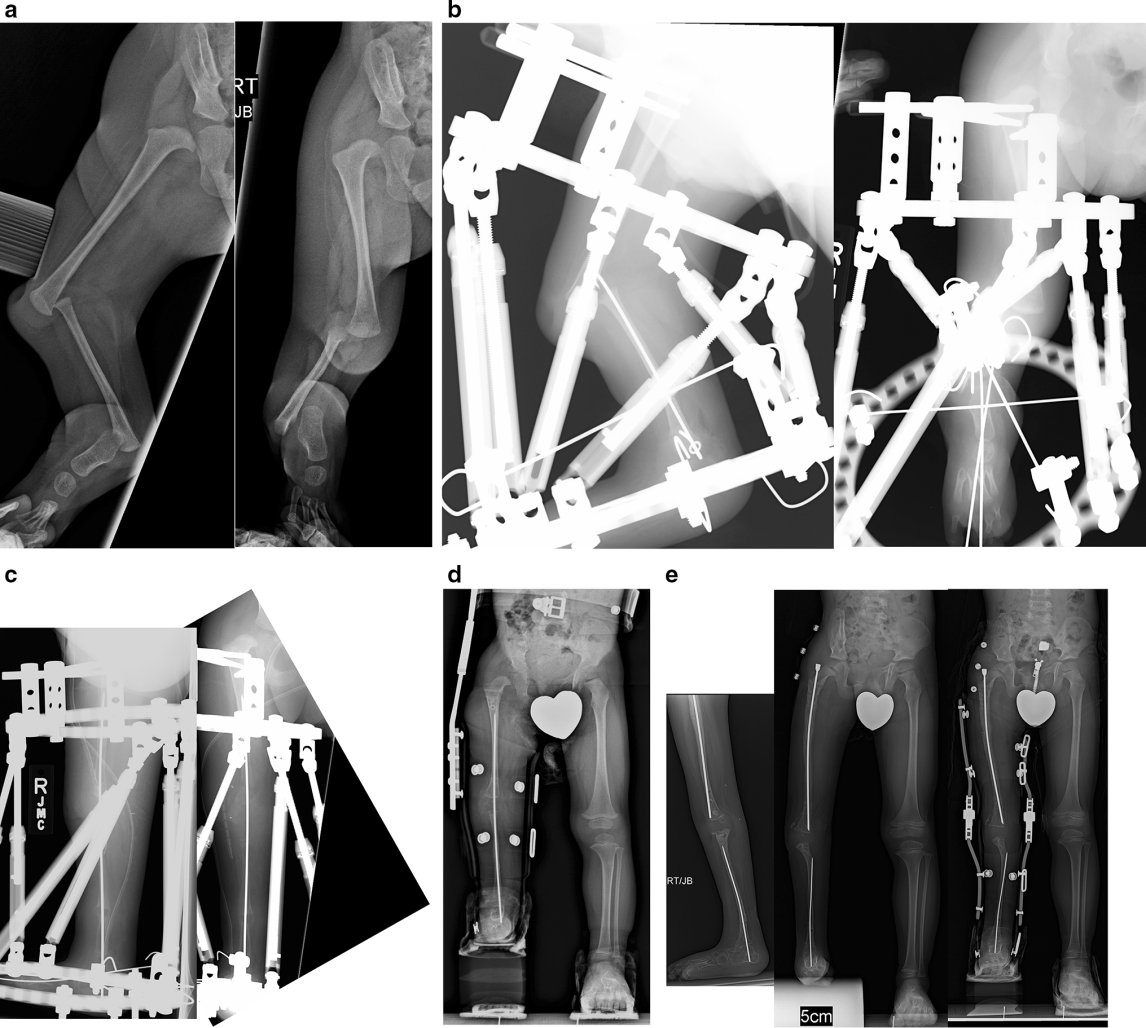
**a** Preoperative AP (*left*) and lateral (*right*) radiographs of the right leg of a 15-month-old boy born with unilateral tibial hemimelia, Paley type 5C. There is no patella or quadriceps muscle. The knee and ankle have severe contractures. **b** Lateral (*left*) and AP (*right*) radiographs showing TSF in place with one ring on femur and one on the foot. There is a temporary epiphysiodesis wire in the fibula hooked at both ends. There is a transverse wire through the fibula to transport it distally. **c** Intraoperative (hemovac seen) lateral (*left*) and AP (*right*) radiographs after complete correction of the knee and ankle and after the second staged surgery to fuse the ankle and to temporarily arthrodese the knee using an axial wire. Tendon transfers were also done to replace the absent quadriceps muscle. **d** AP standing radiograph following removal of external fixator 3 months later. The axial wire was left in place to protect the knee for 6 more months. There is excellent alignment with significant leg length difference. **e** Standing lateral (*left*) and AP (*middle*, *right*) radiographs at age 5 yers, after lengthening of the femur and tibia and after a pelvic osteotomy was done to stabilize the hip. Separate intramedullary wires remain in place to protect the bones from fracture and to guide hypertrophy. A KAFO is used for several years—until the knee becomes stable—to allow knee flexion and extension while protecting varus and valgus bending on the hypertrophying knee joint for several years until the knee becomes stable

It may take up to 5–6 months to centralize the knee and ankle such that the contractures at the knee and ankle are eliminated, the proximal fibula is centered under the femur and the talus is centered under the fibula. Once this is accomplished, the patient returns to the operating room where the distal ring and wires are removed and the leg is prepped and draped free. Tegaderm dressing is placed over the wire sites at the foot and the upper femoral ring after the patient is prepped and covered with a towel to prevent contact with the more sterile operative field. A transverse concave proximal incision is made at the level of the knee. The peroneal nerve is liberated and decompressed from the fibula. The biceps tendon is detached from the fibula laterally and the semitendinosis muscle is dissected free medially. The tensor fascia lata and its iliotibial band are also detached and mobilized. These three muscles are later connected to the remnant of the quadriceps muscle (usually ends in the mid thigh and does not continue to the knee) if present and then prepared to be transferred to the fibula to act as quadriceps muscle. The transferred muscles are all connected together with the lateral and medial muscles balancing each other and centralizing themselves to the iliotibial band, which acts as a central rib for the connection of all of these muscles. It is eventually attached to the front of the fibula to act as the patellar tendon.

Before connecting the transferred muscle, it is important to release the fibula from the tethering posterior capsule. To avoid injury to the popliteal vessels, the medial head of the gastrocnemius muscle is identified. The vessels can easily be found just lateral to this muscle belly. The posterior capsule can now be safely cut. The next step is to stabilize the fibular head by creating new ligaments to the femur. This is done by creating an interosseous ligament between the femur and the fibula and two collateral ligaments. An allograft anterior or posterior tibial tendon is used. A drill hole is made transversely across the femoral epiphysis. A distal to proximal partial thickness hole is drilled to intersect the transverse drill hole at a T junction. The allograft tendon is split in half for half of its length. Both limbs of the split allograft tendon are inserted retrograde through the distal femoral hole exiting out the medial and lateral sides of the femoral epiphysis. The unsplit portion of the tendon is sutured to the tip of the fibular epiphysis. The lateral and medial halves are then pulled tight and advanced distally to be sutured to the medial and lateral aspects of the fibula. These form the medial and lateral collateral ligaments with a central cruciate-like ligament inside the joint. The fibula is now stabilized and tethered to the femur but is free to flex and extend 90°. Dislocation and recurrent flexion deformity were the main reason for failure of the original Brown procedure. The interosseous ligaments prevent subluxation from occurring while permitting hinge flexion of the two bones. These ligaments help load transfer between the femur and fibula in order to promote hypertrophy of the joint. The muscle transfers can now be sutured to the fibula to substitute for the absent quadriceps muscle. The ankle is fused as previously described. A wire is inserted from the foot through the fibula. The retrograde fibular wires stop at the knee. After the incision is closed, the foot ring, which was removed at the start of the procedure, is reapplied with the circular fixator struts. The foot is immobilized in a plantigrade position and the knee in full extension. One transverse fibular wire is inserted to compress the ankle fusion and to slightly distract the knee. Hinges are placed at the knee to allow the knee to be ranged passively in therapy and at home. The knee is always locked in extension during walking and at rest so as to allow the transferred muscles to heal. An alternative and older method was to advance the fibular wire across the knee to allow the ligaments, capsule and transferred muscles to heal before moving the knee. This wire would be removed 6 months later. The external fixator is left in situ for 3 months and then removed. At the time of removal it is sometimes necessary to cut the fascia lata at the level of the greater trochanter if an abduction contracture has developed. A cast is used for 1 month and then an HKAFO is prepared. While amputation remains the gold standard for treatment of Paley type 5c cases, this type of reconstruction should be considered on one side of bilateral type 5c cases or when amputation is not an acceptable option for the patient. Knee fusion is the other alternative to amputation. To save length, knee fusion could be done instead of the Paley knee reconstruction technique described above. The proximal fibular epiphysis should be fused to the distal femoral epiphysis in a way so as not to damage the adjacent physes of these two bones. This is achieved by minimizing dissection of the epiphyses so as not to devascularize them and by cutting across to the ossific nuclei of both bones. The ossific nuclei can be held in apposition to each other using the external fixator and an intramedullary wire until the knee is fused.

## Conclusion

Tibial hemimelia can be best classified with the Paley classification. This classification is based on progressive deficiency and pathoanatomy and as such serves to guide reconstructive options. Combining this better understanding of the patho-anatomy of tibial hemimelia subtypes with improved distraction/surgical techniques offers new improved reconstructive options for all types of tibial hemimelia.

Through-knee amputation remains the most commonly used procedure for complete tibial aplasia (Paley type 5 tibial hemimelia). Amputations are reliable options that can be carried out by most orthopedic surgeons. The presence of a patella has now been shown to greatly improve the prognosis of treatment even for complete tibial aplasia [48, 60, 63]. Staged reconstruction using distraction by the Paley technique should also be considered, at least on one side, in bilateral cases of Paley types 5b and 5c.

Amputation is likely overused in Paley types 2, 3, and 4 tibial hemimelia. Reconstructive results for these types using the newer techniques described herein are reliable and successful in achieving a functional lower extremity. It is important to remember that a stable, painless plantigrade stiff ankle or ankle fusion is very functional and has sensation and proprioception. No prosthetic foot provides sensation or proprioception.

The reconstructive options for tibial hemimelia have improved greatly over the past three decades. With continued success and improvements, they may one day overtake the amputation option.
